# Taxonomy and Phylogenetic Appraisal of Dothideomycetous Fungi Associated with *Magnolia*, *Lilium longiflorum* and *Hedychium coronarium*

**DOI:** 10.3390/jof8101094

**Published:** 2022-10-17

**Authors:** Nimali I. de Silva, Kasun M. Thambugala, Danushka S. Tennakoon, Samantha C. Karunarathna, Jaturong Kumla, Nakarin Suwannarach, Saisamorn Lumyong

**Affiliations:** 1 Research Center of Microbial Diversity and Sustainable Utilization, Faculty of Science, Chiang Mai University, Chiang Mai 50200, Thailand; 2 Department of Biology, Faculty of Science, Chiang Mai University, Chiang Mai 50200, Thailand; 3 Genetics and Molecular Biology Unit, Faculty of Applied Sciences, University of Sri Jayewardenepura, Gangodawila, Nugegoda 10250, Sri Lanka; 4 Center for Yunnan Plateau Biological Resources Protection and Utilization, College of Biological Resource and Food Engineering, Qujing Normal University, Qujing 655011, China; 5 Academy of Science, The Royal Society of Thailand, Bangkok 10300, Thailand

**Keywords:** 3 new species, *Dothideomycetes*, multi-locus sequence analysis, saprobes, taxonomy

## Abstract

This paper highlights the taxonomy of some interesting saprobic microfungi associated with dead plant materials of *Hedychium coronarium*, *Lilium longiflorum*, and *Magnolia* species. The taxa reported in this study belong to the orders *Pleosporales* and *Kirschsteiniotheliales* (*Dothideomycetes*). These taxa were identified based on multi-locus phylogeny of nuclear ribosomal DNA (rDNA) (LSU, SSU, and ITS) and protein-coding genes (*tef1-α* and *rpb2*), together with comprehensive morphological characterization. Two novel saprobic species, *Leptoparies magnoliae* sp. nov. and *Neobambusicola magnoliae* sp. nov., are introduced from *Magnolia* species in Thailand. Another new species, *Asymmetrispora zingiberacearum* sp. nov., is also described from dead stems of *H. coronarium*, which is the first asexual morph species of the genus *Asymmetrispora.* In addition, *Ramusculicola thailandica* and *Kirschsteiniothelia thailandica* are reported as new host records from dead twigs of *Magnolia* species. *Sphaerellopsis paraphysata* is reported as a new host record from *L. longiflorum*. Newly described taxa are compared with other similar species and detailed descriptions, micrographs, and phylogenetic trees to show the positions are provided.

## 1. Introduction

The exploration of the tropics to discover and describe fungal life has recently gained immense scientific interest. In this contribution, we investigate novel and existing fungal species and their host plant associations. Fungi play fundamental ecological roles as decomposers, mutualists, and pathogens [1]. They also help carbon cycling and biogeochemical processes and mediate mineral nutrition of plants in forest ecosystems [2,3,4]. Fungi have an important contribution to ecosystems, as higher saprobic fungal diversity intensifies the decomposition process and higher mycorrhizal diversity enhances plant diversity, ecosystem functioning, and nutrient assimilation [5]. Mycologists have proposed different estimations for existing and described fungal species numbers. Hawksworth and Lucking [6] stated that the estimated fungal species ranged from 2.2 to 3.8 million fungal species, and only 120,000 (8%) have been described. This implies that a large number of fungi still remain to be discovered. The possible reasons for the observed discrepancy can be due to fungi being poorly studied in many countries, regions, and host species [7].

Tropical rainforests harbor the highest fungal diversity [3]. Fungi present in tropical regions and their vegetations are not well studied; thereby, fungal novelties might be high [3,8]. A recent study on the fungi of northern Thailand showed that up to 96% of species found in Thailand are new to science [4]. Different factors affect the diversity and distribution of fungi, such as biotic factors viz. host plant species, dispersal mechanisms, and competition among different fungal species and abiotic factors such as seasonal changes, temperature, humidity, and nutrient availability [9,10]. Plant diversity and their physiological factors greatly influence fungal community structure by providing diverse microclimates, complex habitats, and different organic substrates [5]. The fungal mycelial growth can be influenced by the area, chemistry, and available volume of the substrate [11]. On the other hand, considering abiotic factors, high relative humidity is essential for the growth and sporulation of fungi [9]. Precipitation elevates the growth of the fungi; thereby, they densely colonize on decaying plant debris [11]. Further, the diversity and composition of a fungal community are thought to be influenced by nitrogen availability, atmospheric CO_2_ concentration, resource supply, and soil depth [12,13,14]. In addition, fungal adaptations and their interactions with plants and the environment also have a greater influence on the diversity and richness of fungal species [9]. Fungi that inhabit tropical regions possess different morphological adaptations such as ascospores with appendages or sheaths, which might help them to disperse to different niches or habitats [9]. Costa and Gusmão [11] investigated *Pestalotiopsis* spp. from leaf litter in the Atlantic Forest only during the wet season. They explained that the characteristic feature of *Pestalotiopsis* spp. bearing terminal appendages can adhere to a surface during high humidity. These distinct morphological features are important for providing attachment and effective dispersal of spores during heavy tropical rains [9]. In contrast, Costa and Gusmão [11] found *Guignardia* spp. only during the dry season because spores of *Guignardia* spp. disperse with the aid of air currents over long distances. During rainy seasons, the dispersal of *Guignardia* spp. is affected by rainfall [11]. These facts indicate that fungi show great variation in morphology for reproduction, effective dispersal, and continuing their life cycle.

In the current study, we studied *Pleosporales* Luttr. ex M.E. Barr and *Kirschsteiniotheliales* Hern.-Restr., R.F. Castañeda, Gené, and Crous fungi associated with dead plant materials of *H. coronarium*, *L. longiflorum*, and *Magnolia* species. *Pleosporales* is the largest order in the *Dothideomycetes* Erikss. and K. Winka [15]. *Pleosporales* was established by Barr [16] based on the family *Pleosporaceae* Nitschke with the species type *Pleospora herbarum* (Pers.) Rabenh. [17]. Hongsanan et al. [18] accepted 91 families in *Pleosporales* based on morphology and phylogenetic evidence. Further, they stated that the divergence time for *Pleosporales* was 205 MYA (stem age). Pleosporalean fungi have a cosmopolitan distribution. They exhibit as epiphytes, endophytes, and pathogens (living leaves or stems, hyperparasites on fungi or insects). Some of them can be lichenized or are saprobes of dead plant stems, leaves, or bark [15]. *Pleosporales* fungi have asexual and sexual morphs. The sexual morph is characterized by perithecioid ascomata with a papillate apex, comprising bitunicate, fissitunicate asci, and various shapes of ascospores with different pigmentation, septation, and with or without a gelatinous sheath [15,18]. Meanwhile, asexual morphs can be either coelomycetous or hyphomycetous. Generally, *Phoma* or phoma-like asexual morphs are common among pleosporalean species [15]. In this study, we focus on some interesting pleosporalean fungi belonging to *Leptosphaeriaceae* M.E. Barr, *Lophiostomataceae* Sacc., *Sulcatisporaceae* Kaz. Tanaka and K. Hiray., and *Teichosporaceae* M.E. Barr. In addition, we observed hyphomycetous fungi belonging to *Kirschsteiniotheliales*. *Kirschsteiniotheliales* was established by Hernandez et al. [19]. The family type is *Kirschsteiniotheliaceae* Boonmee and K.D. Hyde, and the genus type is *Kirschsteiniothelia* D. Hawksw [19]. In addition to that, this order consists of *Brachysporiella* Bat. and *Taeniolella* S. Hughes genera *incertae sedis* of *Kirschsteiniotheliales* [18]. Hongsanan et al. [18] estimated the divergence time of *Kirschteiniotheliales* as 221 MYA. Members of *Kirschsteiniotheliales* are mostly saprobes on dead wood in terrestrial and aquatic habitats [18,19]. The aims of this study are to identify and describe interesting saprobic fungi associated with *H. coronarium*, *L. longiflorum*, and *Magnolia* species. Herein, we provide full descriptions, color plates, and phylogenetic trees to show the positions of the novel taxa and new host records.

## 2. Materials and Methods

### 2.1. Samples Collection, Morphological Studies, and Isolation

Dead twigs attached to *Magnolia* spp. in Chiang Mai Province, Thailand, were collected in this study. Dead stems of *H. coronarium* and leaf litter from *L. longiflorum* in Taiwan Province of China were also collected in this study. These plant specimens were transferred to the laboratory and examined with a JNOEC JSZ4 stereomicroscope. Morphological characteristics were observed using an OLYMPUS SZ61 compound microscope. Photographs of morphological characters were captured with a Canon EOS 600D digital camera mounted on a Nikon ECLIPSE 80i compound microscope. The Tarosoft (R) image framework v. 0.9.0.7 was used to measure all microscopic measurements. The Adobe Photoshop CS3 Extended version was used to process photographs further. Pure fungal cultures were isolated following the protocol described in Senanayake et al. [20]. Germinating ascospores and conidia were transferred aseptically to potato dextrose agar (PDA) for further cultural and molecular analyses. Culture characteristics of pure fungal cultures, such as growth rate and colony characteristics, were observed and recorded at room temperature (25 °C).

The collections made in the current study were deposited at the Mae Fah Luang University Herbarium (Herb. MFLU), Chiang Rai Province, Thailand, the herbarium of Cryptogams, Kunming Institute of Botany Academia Sinica (HKAS), Kunming, Yunnan Province, China, and National Chiayi University Herbarium (NCYU). The living fungal cultures recovered in this study were deposited at Mae Fah Luang University Culture Collection (MFLUCC) and National Chiayi University Culture Collection (NCYUCC). Faces of Fungi numbers and Index Fungorum numbers were registered as described in Jayasiri et al. [21] and Index Fungorum [22], respectively.

### 2.2. DNA Extraction and PCR Amplification

Genomic DNA was extracted using one-week-old fungal cultures on PDA [23]. The mycelia were scraped off from pure cultures, and genomic DNA was extracted using Biospin fungus genomic DNA kit (BioFlux^®^, Hangzhou, China) according to the manufacturer’s instructions. In addition, genomic DNA was extracted from the fruiting bodies on the natural substrate of *Leptoparies magnoliae* (MFLU 18-1291), *Neobambusicola magnoliae* (HKAS 107122), and *Ramusculicola thailandica* (HKAS 107136) using a DNA extraction kit (BioFlux^®^, Hangzhou, China) according to the manufacturer’s instructions. The DNA products were kept at 4 °C for DNA amplification and maintained at −20 °C for long-term storage.

Selected genes, such as the partial gene regions of Internal Transcribed Spacers (ITS) and 28 S ribosomal RNA (LSU), 18 S ribosomal RNA (SSU), and Translation Elongation Factor 1–alpha (*tef1-α*), were amplified using appropriate primers via polymerase chain reaction (PCR). The LSU region was amplified with primer pair LR0R and LR5 [24]. The SSU region was amplified with primer pair NS1 and NS4, and the ITS region was amplified with primer pair ITS5 and ITS4 [25]. The part of the *tef1-α* region was amplified with primer pair EF1-983F and EF1-2218R [26]. The total volume of the final PCR mixture was 25 μL, which was composed of 1 μL of DNA template, 1 μL of each forward and reverse primer, 12.5 μL of 2×Easy Taq PCR SuperMix (a mixture of EasyTaq TM DNA Polymerase, dNTPs, and optimized buffer, Beijing TransGen Biotech Co., Ltd., Beijing, China), and 9.5 μL of ddH_2_O. PCR amplification of LSU, SSU, ITS, and *tef1-α* included an initial denaturing step of 94 °C for 3 min., followed by 40 amplification cycles of 94 °C for 45 s, 55 °C for 50 s, and 72 °C for 1 min. and a final extension step of 72 °C for 10 min.

PCR purification and sequencing of amplified PCR products were conducted at Shanghai Sangon Biological Engineering Technology & Services Co., Ltd., Shanghai, China.

Sequences of the individual genes were aligned with MAFFT v. 7 online version [27] with default settings. The alignments were manually improved where necessary and to exclude incomplete portions at the ends of the sequences before the analyses using BioEdit v. 7.0.5.2 [28]. The newly generated sequences in this study were deposited in GenBank, and accession numbers were mentioned in relevant entries. Details of the sequences used for phylogenetic analysis are provided in supplementary documents (Tables S1–S4).

### 2.3. Molecular Phylogenetic Analyses

Maximum likelihood (ML) and Bayesian inference (BI) were used to estimate phylogenetic relationships. ML analysis was performed using RAxML GUI v. 1.3 [29]. Evolutionary models for phylogenetic analyses were selected independently for each locus using MrModeltest v. 3.7 [30] under the Akaike Information Criterion (AIC). Parameters for ML were set to rapid bootstrapping, and the analysis was conducted for 1000 replicates using the GTR + GAMMA model of nucleotide substitution. Bayesian analysis was conducted with MrBayes v. 3.1.2 [31]. The GTR + I + G was selected as the best-fit nucleotide substitution model for *Leptosphaeriaceae*, *Lophiostomataceae*, *Sulcatisporaceae*, *Teichosporaceae*, and *Kirschsteiniothelia.* Parameters of BI in MrBayes v. 3.2: Markov chains were set to run 1,000,000 generations, resulting trees were sampled every 100th generation (printfreq = 100), and 10,000 trees were obtained. Initial trees were discarded (20% burn-in value), and the remaining trees were used to evaluate posterior probabilities (PP) in the majority rule consensus tree. These resulting trees from ML and BI were visualized with FigTree v1.4.0 [32] and edited in Microsoft PowerPoint (2010).

## 3. Results

### 3.1. Phylogenetic Relationships

#### 3.1.1. *Pleosporales*

##### *Leptosphaeriaceae* LSU, SSU, and ITS Phylogeny

**Figure 1:**
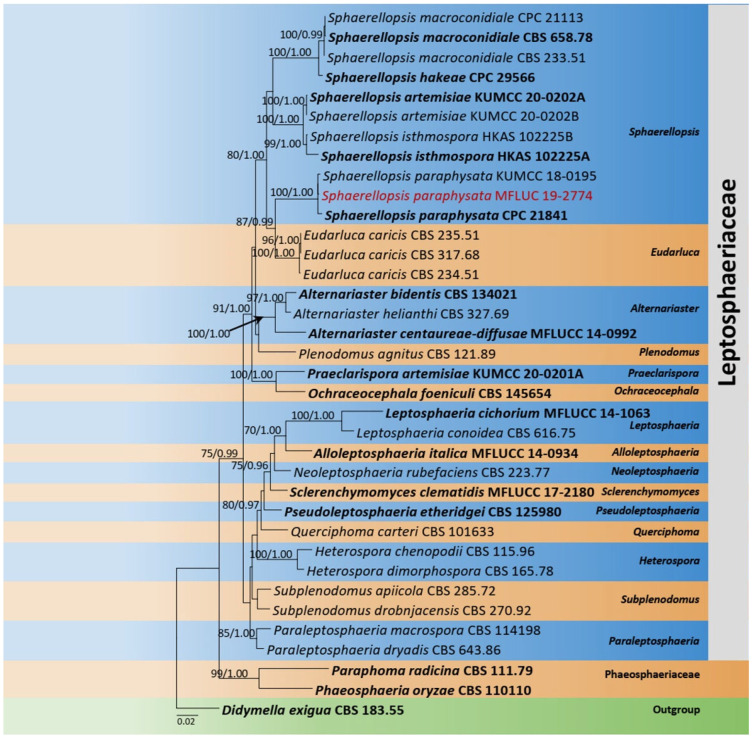
Phylogram generated from maximum likelihood analysis is based on combined LSU, SSU, and ITS sequence data. Related sequences of *Leptosphaeriaceae* were obtained from Doilom et al. [33]. ML bootstrap values equal to or greater than 75% and Bayesian posterior probabilities (BYPP) equal to or greater than 0.95 are indicated above the branches. The tree was rooted to *Didymella exigua* (CBS 183.55). The newly generated sequences are indicated in red. Type and ex-type strains are in bold.

The combined dataset of LSU, SSU, and ITS comprised 36 strains, representing *Leptosphaeriaceae* with *Didymella exigua* (Niessl) Sacc. (CBS 183.55) as the outgroup taxon. The topology of the resulting phylogram of maximum likelihood analysis is largely similar to Bayesian analysis. The combined gene analyses comprised 2400 characters after alignment (900 characters for LSU, 1000 characters for SSU, and 500 characters for ITS). The best RAxML tree with a final likelihood value of −10,207.279983 is presented. The matrix had 546 distinct alignment patterns, with 26.03% undetermined characters or gaps. Estimated base frequencies were as follows: A = 0.247348, C = 0.219380, G = 0.270676, T = 0.262596; substitution rates AC = 1.898935, AG = 3.718119, AT = 2.835578, CG = 0.694011, CT = 6.921459, GT = 1.000000; gamma distribution shape parameter *α* = 0.571073. Eleven strains of *Sphaerellopsis* Cooke were clustered together in a monophyletic clade. The new strain *Sphaerellopsis paraphysata* (MFLU 19-2774) was clustered with the ex-type *S. paraphysata* (CPC 21841) with 100% ML and 1.00 BYPP statistical supports (Figure 1).

##### *Lophiostomataceae* SSU, ITS, LSU, *tef1-α*, and *rpb2* Phylogeny

**Figure 2:**
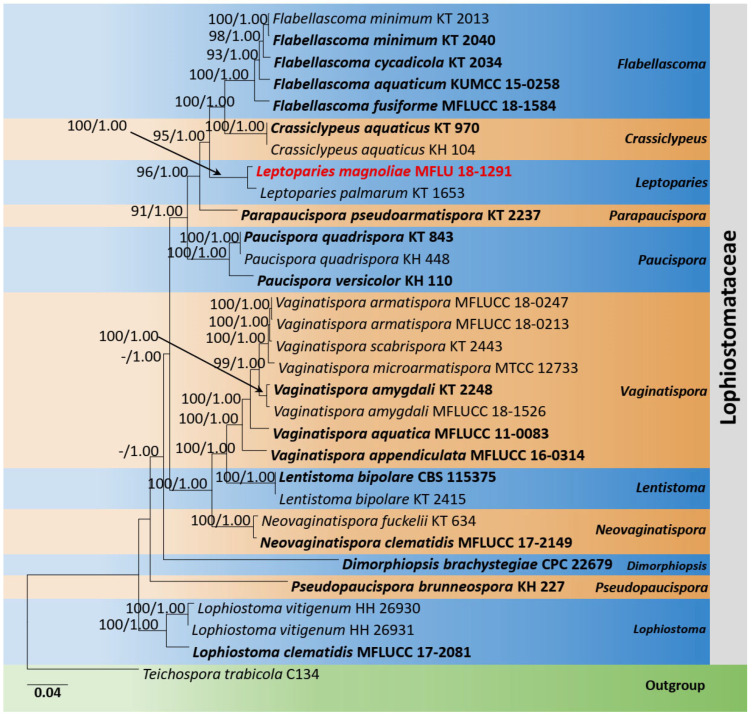
Phylogram generated from maximum likelihood analysis is based on combined LSU, SSU, ITS, *tef1-α*, and *rpb2* sequence data. Related sequences of *Leptoparies* and closely related genera in *Lophiostomataceae* were obtained from Andreasen et al. [34]. ML bootstrap values equal to or greater than 75% and Bayesian posterior probabilities (BYPP) equal to or greater than 0.95 are indicated above the branches. The tree was rooted to *Teichospora trabicola* (C134). The newly generated sequences are indicated in red. Type and ex-type strains are in bold.

The phylogenetic analyses of *Lophiostomataceae* were constructed based on SSU, ITS, LSU, *tef1-α*, and *rpb2* sequence data. Thirty-one strains are included in the combined gene analyses, comprising 4590 characters after alignment (970 characters for SSU, 520 characters for ITS, 1200 characters for LSU, 900 characters for *tef1-α*, and 1000 characters for *rpb2*). *Teichospora trabicola* Fuckel (C134) is used as the outgroup taxon. The best RAxML tree with a final likelihood value of −21,327.935224 is presented. The matrix had 1323 distinct alignment patterns, with 25.67% undetermined characters or gaps. Estimated base frequencies were as follows: A = 0.248907, C = 0.247867, G = 0.268967, T = 0.234259; substitution rates AC = 1.542394, AG = 3.667797, AT = 1.446407, CG = 1.625869, CT = 9.070211, GT = 1.000000; gamma distribution shape parameter *α* = 0.405551. *Leptoparies* formed a distinct and well-supported lineage in *Lophiostomataceae*. The genus comprises only one species, *L. palmarum* (KT1653). The current phylogeny showed a new strain (MFLU 18-1291) clustered sister to *L. palmarum* (KT1653) (Figure 2).

##### *Sulcatisporaceae* LSU, ITS, SSU, and *tef1-α* Phylogeny

**Figure 3:**
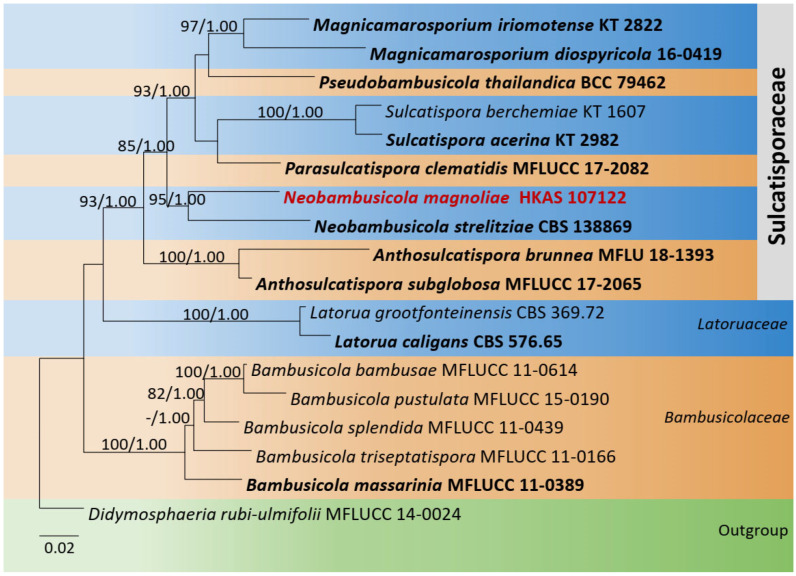
Phylogram generated from maximum likelihood analysis is based on combined LSU, SSU, ITS, and *tef1-α* sequence data. Related sequences of *Sulcatisporaceae* were obtained from Phukhamsakda et al. [35,36]. ML bootstrap values equal to or greater than 75% and Bayesian posterior probabilities (BYPP) equal to or greater than 0.95 are indicated above the branches. The tree was rooted to *Didymosphaeria rubi-ulmifolii* (MFLUCC 14-0024). The newly generated sequences are indicated in red. Type and ex-type strains are in bold.

The combined dataset of LSU, ITS, SSU, and *tef1-α* comprised 18 strains, representing *Bambusicolaceae* D.Q. Dai and K.D. Hyde, *Sulcatisporaceae*, and *Latoruaceae* Crous with *Didymosphaeria rubi-ulmifolii* Ariyaw., Camporesi, and K.D. Hyde (MFLUCC 14-0024) as the outgroup taxon. The topology of the resulting phylogram of maximum likelihood analysis is largely similar to Bayesian analysis. The combined gene analyses comprised 3500 characters after alignment (1000 characters for LSU, 1000 characters for SSU, 550 characters for ITS, and 950 characters for *tef1-α*). The best RAxML tree with a final likelihood value of −13,636.126780 is presented. The matrix had 906 distinct alignment patterns, with 42.30% undetermined characters or gaps. Estimated base frequencies were as follows: A = 0.237741, C = 0.249071, G = 0.276720, T = 0.236467; substitution rates AC = 1.032758, AG = 1.719261, AT = 0.851641, CG = 0.787256, CT = 4.449475, GT = 1.000000; gamma distribution shape parameter *α* = 0.534809. *Neobambusicola* formed a distinct and well-supported clade in *Sulcatisporaceae*. The new species *Neobambusicola magnoliae* (MFLU 18-1291) formed a monophyletic clade with *N. brunnea* (MFLU 18-1393) and *N. strelitziae* (CBS 138869) (Figure 3).

##### *Teichosporaceae* LSU, ITS, SSU, *tef1-α*, and *rpb2* Phylogeny

**Figure 4:**
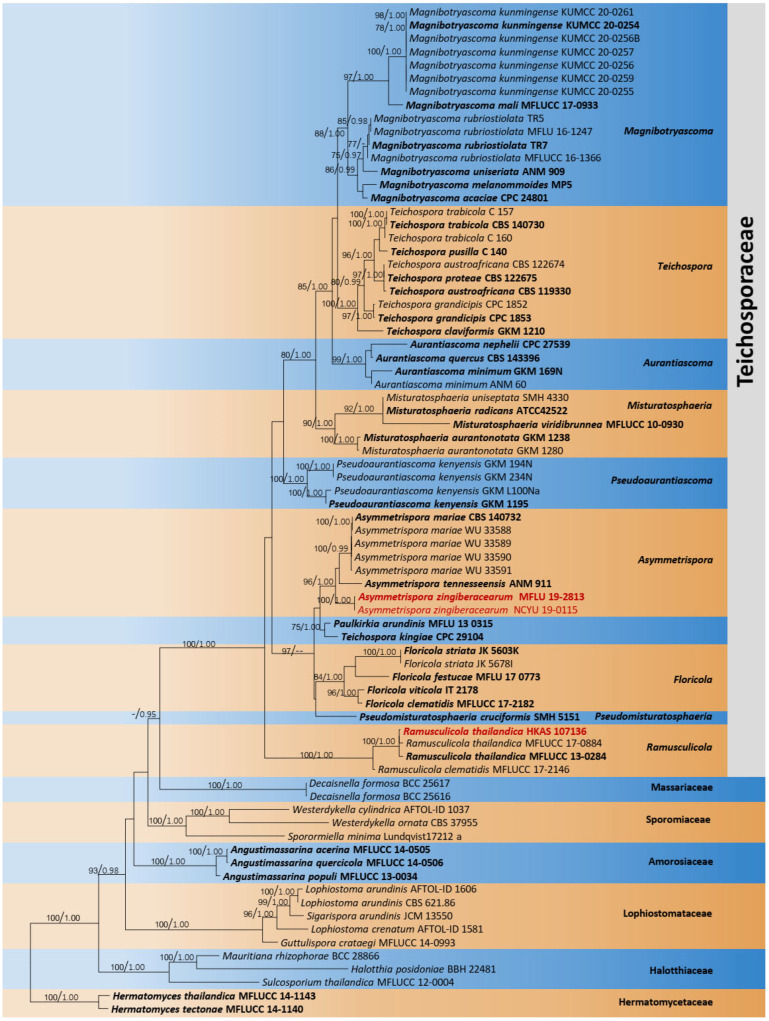
Phylogram generated from maximum likelihood analysis is based on combined LSU, ITS, SSU, *tef1-α*, and *rpb2* sequence data. Related sequences of *Teichosporaceae* were obtained from Tennakoon et al. [37]. ML bootstrap values equal to or greater than 75% and Bayesian posterior probabilities (BYPP) equal to or greater than 0.95 are indicated above the branches. The tree was rooted to *Hermatomyces tectonae* (MFLUCC 14-1140) and *H. thailandica* (MFLUCC 14-1143). The newly generated sequences are indicated in red. Type and ex-type strains are in bold.

The combined dataset of LSU, ITS, SSU, *tef1-α*, and *rpb2* comprised 76 strains, representing *Teichosporaceae* with *Didymella exigua* (CBS 183.55) as the outgroup taxon. The topology of the resulting phylogram of maximum likelihood analysis is largely similar to Bayesian analysis. The combined gene analyses comprised 4200 characters after alignment (900 characters for LSU, 1000 characters for SSU, 500 characters for ITS, 900 characters for *tef1-α*, and 900 characters for *rpb2*). The best RAxML tree with a final likelihood value of −24,166.180022 is presented. The matrix had 1659 distinct alignment patterns, with 43.60% undetermined characters or gaps. Estimated base frequencies were as follows: A = 0.242860, C = 0.254703, G = 0.275172, T = 0.227265; substitution rates AC = 1.277952, AG = 3.342937, AT = 1.743291, CG = 1.124388, CT = 8.356486, GT = 1.000000; gamma distribution shape parameter *α* = 0.502544. Eight strains of *Asymmetrispora* Thambug. and K.D. Hyde formed a monophyletic clade sister to *Paulkirkia arundinis* Wijayaw., Wanas., Tangthir., Camporesi, and K.D. Hyde (MFLU 13-0315). Two strains of the new species, *Asymmetrispora zingiberacearum* (MFLU 19-2813) and (NCYU 19-0115), formed a distinct lineage within *Asymmetrispora* (Figure 4).

#### 3.1.2. Kirschteiniotheliales

##### *Kirschsteiniothelia* LSU, SSU, and ITS Phylogeny

**Figure 5:**
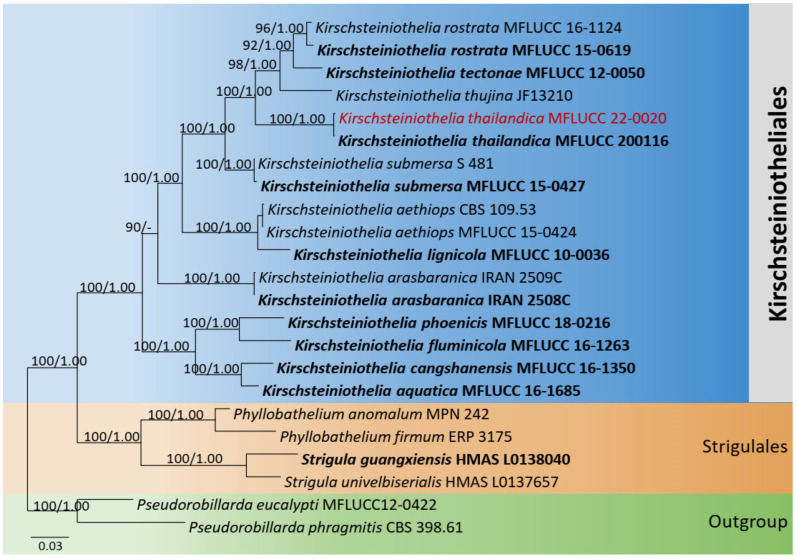
Phylogram generated from maximum likelihood analysis is based on combined LSU, SSU, and ITS sequence data. Related sequences of *Kirschsteiniothelia* species were obtained from Sun et al. [38]. ML bootstrap values equal to or greater than 75% and Bayesian posterior probabilities (BYPP) equal to or greater than 0.95 are indicated above the branches. The tree was rooted to *Pseudorobillarda eucalypti* (MFLUCC 12-0422) and *P. phragmitis* (CBS 398.61). The newly generated sequences are indicated in red. Type and ex-type strains are in bold.

The combined dataset of LSU, SSU, and ITS comprised 23 strains, representing *Kirschsteiniothelia* species (*Kirschsteiniotheliales*) and *Strigulales* Lücking, M.P. Nelsen, and K.D. Hyde with *Pseudorobillarda eucalypti* Tangthir. and K.D. Hyde (MFLUCC 12-0422) and *P. phragmitis* (Cunnell) M. Morelet (CBS 398.61) as the outgroup taxa. The topology of the resulting phylogram of maximum likelihood analysis is largely similar to Bayesian analysis. The combined gene analyses comprised 2530 characters after alignment (1000 characters for LSU, 1000 characters for SSU, and 530 characters for ITS). The best RAxML tree with a final likelihood value of −12,923.087464 is presented. The matrix had 1038 distinct alignment patterns, with 38.97% undetermined characters or gaps. Estimated base frequencies were as follows: A = 0.237143, C = 0.243017, G = 0.296383, T = 0.223457; substitution rates AC = 1.037499, AG = 2.061321, AT = 0.808903, CG = 1.068397, CT = 4.800710, GT = 1.000000; gamma distribution shape parameter *α* = 0.601418. Seventeen strains of *Kirschsteiniothelia* clustered together in a monophyletic clade. The new strain (MFLUCC 22-0020) clustered with the ex-type *K. thailandica* Y.R. Sun, Yong Wang bis, and K.D. Hyde (MFLUCC 20-0116) with 100% ML and 1.00 BYPP statistical supports (Figure 5).

### 3.2. Taxonomy

**Phylum *Ascomycota*** Caval.-Sm.

**Class *Dothideomycetes*** O.E. Erikss. and Winka

**Subclass *Pleosporomycetidae*** Schoch et al.

***Pleosporales*** Luttr. ex M.E. Barr

***Leptosphaeriaceae*** M.E. Barr, Mycotaxon 29: 503 (1987)

Type: *Leptosphaeria* Ces. and De Not., Comm. Soc. crittog. Ital. 1(fasc. 4): 234 (1863)

Notes: Barr [16] established *Leptosphaeriaceae* (*Pleosporales*, *Dothideomycetes*) with the type *Leptosphaeria*. The sexual morph is characterized by immersed, erumpent to superficial ascomata, scleroplectenchymatous peridium, cylindrical asci, and hyaline to brown, transversely septate ascospores. The asexual morph is coelomycetous or hyphomycetous [39,40,41]. *Leptosphaeriaceae* species exhibit as saprobes, hemibiotrophs, pathogens, or parasites. Members of this family are found on leaves and stems of herbaceous or woody plants in terrestrial and aquatic habitats [39,42,43]. Hongsanan et al. [18] accepted 14 genera in this family.

***Sphaerellopsis*** Cooke, Grevillea 12 (6): 23 (1883)

Type: *Sphaerellopsis quercuum* Cooke, Grevillea 12 (no. 61): 23 (1883)

Notes: *Sphaerellopsis* was introduced by Cooke [44] and typified with *S. quercuum* Cooke. Subsequently, this genus was re-circumscribed by Trakunyingcharoen et al. [45] based on both morphology and phylogeny. *Sphaerellopsis* species have been recorded as saprobic, pathogenic, or mycoparasitic on herbaceous or woody plants in terrestrial habitats [45,46,47]. Currently, there are eight *Sphaerellopsis* species listed in Species Fungorum [48], viz. *S. anomala*, *S. artemisiae*, *S. filum*, *S. hakeae*, *S. isthmospora*, *S. macroconidialis*, *S. paraphysata*, and *S. quercuum*.

***Sphaerellopsis paraphysata*** Crous and Alfenas, IMA Fungus 5(2): 411 (2014)

**Figure 6:**
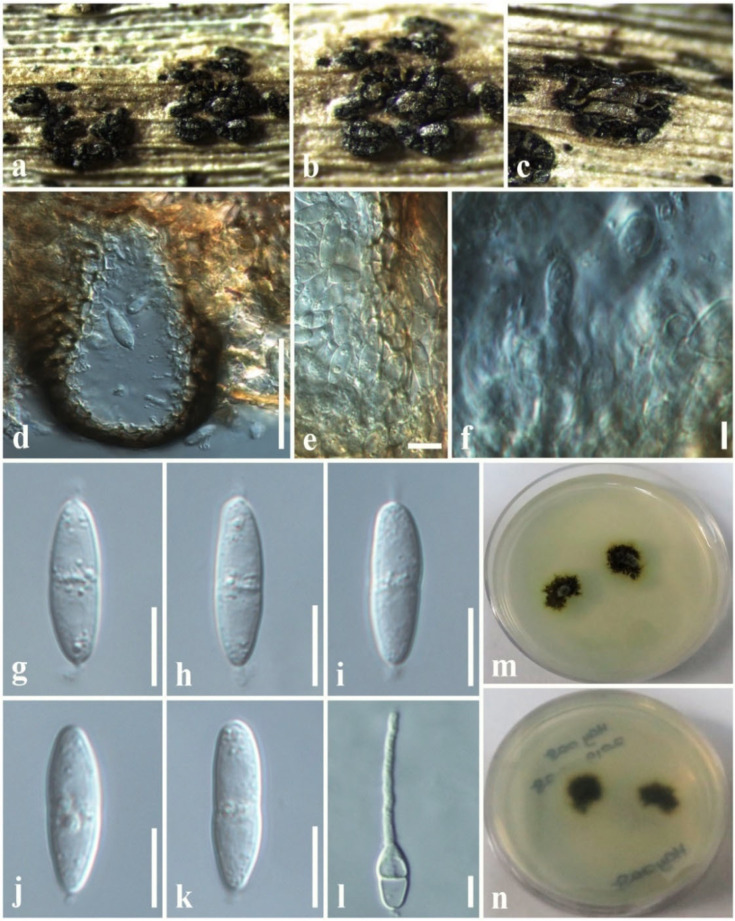
*Sphaerellopsis paraphysata* (MFLU 19-2774, new host record). (**a**,**b**) Conidiomata on host. (**c**) Close-up of conidiomata on host. (**d**) Section through conidioma. (**e**) Conidiomatal wall. (**f**) Conidiogenous cells with developing conidia. (**g**–**k**) Conidia. (**l**) A germinating conidium. (**m**) Colony from above (on PDA). (**n**) Colony from below (on PDA). Scale bars: (**d**) = 50 µm, (**e**,**f**) = 5 µm, (**g**–**l**) = 8 µm.

Index Fungorum Number: IF810844; Faces of fungi number: FoF 04968, Figure 6.

*Associated* with dead leaves of *Lilium longiflorum*. Sexual morph: Undetermined. Asexual morph: Coelomycetous. *Conidiomata* 100–150 × 70–120 μm diam. ($\bar{x}$ = 110 × 80 µm, *n* = 10), black, pycnidial, clustered, semi-immersed to superficial, globose to subglobose, multiloculate, glabrous, ostiole central. *Conidiomata wall* 8–15 μm wide, thin-walled with equal thickness, composed of several layers of hyaline, pseudoparenchymatous cells, arranged in a *textura angularis*. *Conidiophores* reduced to conidiogenous cells. *Conidiogenous cells* 3–6 × 2–4 μm ($\bar{x}\;$ = 4.5 × 3.2 µm, *n* = 20), enteroblastic, phialidic, discrete, determinate, cylindrical to ampulliform to doliiform, hyaline, smooth, thin-walled. *Conidia* 15–17 × 3.5–5 ($\bar{x}$ = 16.5 × 4.5 µm, *n* = 40) μm, hyaline, fusiform to ellipsoidal, mostly one-septate, slightly constricted at the central septum, widest in the middle, with mucilaginous appendages at both ends, smooth-walled.

Culture characteristics: *Colonies* on PDA, 15–20 mm diam. after three weeks at 25 °C; colonies from above: medium dense, irregular, slightly raised, surface smooth with undulate edge, with smooth aspects, yellowish brown at the margin, dark grey to dark brown in the center; reverse: yellowish brown at the margin, dark brown to black in the centre, mycelium dark grey to brown.

Known hosts and distribution: on rusts on *Pennisetum* sp. in Brazil, *Ravenelia macowania* in South Africa [45], on *Liriope spicata* in China [47], on dead leaves of *Lilium longiflorum* in Taiwan Province of China [this study].

Material examined: Taiwan Province of China, Chiayi, Ali Shan Mountain, Fanlu Township area, Dahu forest, on dead leaves of *L. longiflorum* (*Liliaceae*), 15 September 2018, D.S. Tennakoon, HAY028 (MFLU 19-2774); living culture, NCYUCC 19-0262.

GenBank numbers: LSU: ON870393; SSU: ON870916; ITS: ON878080.

Notes: *Sphaerellopsis paraphysata* was introduced by Trakunyingcharoen et al. [45] from the *Pennisetum* species in Brazil. The morphological characteristics of our collection (MFLU 19-2774) tally well with the type in having semi-immersed to superficial, globose to subglobose, multiloculate conidiomata and fusiform to ellipsoidal, one-septate, hyaline conidia with mucilaginous appendages at both ends [45]. However, our collection slightly differs from the type of *S. paraphysata* in having smaller conidiomata (70–120 μm vs. 450 μm) and lacking paraphyses [45]. Multi-gene phylogeny (LSU, SSU, and ITS) also indicates that our collection clusters with other *S. paraphysata* isolates in a well-supported clade (100% ML, 1.00 BYPP, Figure 1). Thus, we identify the new isolate as *S. paraphysata* and considered it as a new host record from *L. longiflorum* in Taiwan Province of China.

***Lophiostomataceae*** Sacc. (as ‘*Lophiostomaceae*’), Syll. Fung. (Abellini) 2: 672 (1883)

Type: *Lophiostoma* Ces. and De Not., Comm. Soc. crittog. Ital. 1(4): 219 (1863)

Notes: *Lophiostomataceae* was introduced by Nitschke [49]. The type genus is *Lophiostoma*, and the type species is *L. macrostomum* [49]. Members of this family are generally distributed in temperate regions. Species of this family are saprobes or necrotrophs that grow on herbaceous and woody plants from terrestrial and aquatic habitats including freshwater and marine environments [50,51]. Members of this family can be identified by their coriaceous to carbonaceous ascomata with the slit-like ostiole [42,50,51].

***Leptoparies*** A. Hashim., K. Hiray. and Kaz. Tanaka, Stud. Mycol. 90: 171 (2018)

Type: *Leptoparies palmarum* A. Hashim., K. Hiray., and Kaz. Tanaka, Stud. Mycol. 90: 171 (2018)

Notes: The monotypic genus *Leptoparies* was introduced by Hashimoto et al. [52]. This genus can be distinguished from the other genera in *Lophiostomataceae* by having relatively thinner and non-carbonized peridium composed of rectangular cells and the absence of the surrounding brown hyphae [52]. *Leptoparies* is similar to *Capulatispora* (*Lophiostomataceae*) in having thin peridium and drawn-out sheaths of ascospores. The sexual morph is characterized by immersed, subglobose ascomata, elongated, laterally compressed ostiolar neck, relatively thin peridium composed rectangular cells, hyaline, fusiform, one-septate, ascospores with a narrow bipolar sheath [52].

***Leptoparies magnoliae*** N.I. de Silva and S. Lumyong, sp. nov.

**Figure 7:**
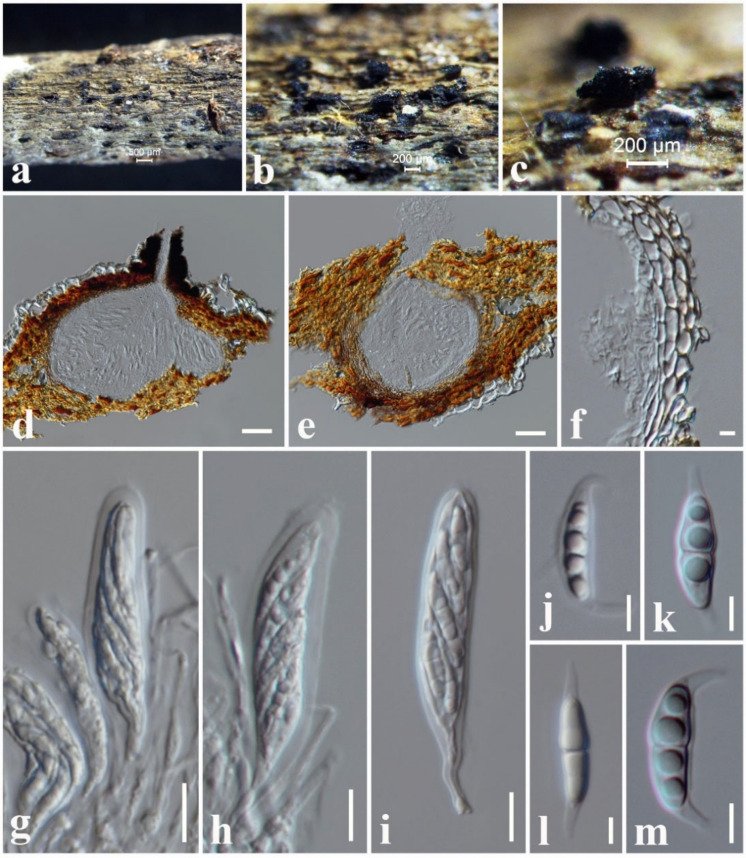
*Leptoparies magnoliae* (MFLU 18-1291, holotype). (**a**) The specimen. (**b**,**c**) Appearance of ascomata on the host substrate. (**d**,**e**) Vertical sections through ascoma. (**f**) Peridium. (**g**,**h**) Pseudoparaphyses and asci. (**i**) Ascus. (**j**–**m**) Ascospores. Scale bars: (**a**) = 500 μm, (**b**,**c**) = 200 μm, (**d**,**e**) = 50 µm, (**g**–**i**) = 10 µm, (**f**,**j**–**m**) = 5 µm.

Index Fungorum number: IF559914, Faces of fungi number: FoF12709, Figure 7.

Etymology: Name reflects the host genus *Magnolia*, from which the new species was isolated.

Holotype: MFLU 18-1291

*Saprobic* on dead twigs attached to *Magnolia* sp. Sexual morph: *Ascomata* 240–290 µm high × 230–270 µm diam. ($\bar{x}$ = 265 × 250 µm, *n* = 10), dark brown to black, solitary or scattered, unilocular, immersed to slightly erumpent, subglobose, ostiolate. *Ostiolar neck* 60–90 µm high × 200–230 µm wide, crest-like, elongated, laterally compressed, composed of globose, brown to black cells, with hyaline periphyses. *Peridium* 15–25 µm wide, composed of several layers of small, brown to dark brown, thin-walled, cells of *textura angularis*, fusing and indistinguishable from the host tissues. *Hamathecium* comprising 1–2 μm wide, numerous, filamentous, indistinct septate, cellular pseudoparaphyses, anastomosing at the apex. *Asci* 40–65 × 8–11 µm ($\bar{x}$ = 58 × 9 µm, *n* = 20), eight-spored, bitunicate, fissitunicate, cylindrical clavate, short pedicellate, apically rounded with an ocular chamber. *Ascospores* 15–19 × 4–6 µm ($\bar{x}$ = 16 × 5 µm, *n* = 30) without sheath, overlapping, uni- to bi-seriate, hyaline, fusiform with obtuse ends, slightly constricted at the septum, smooth, guttulate, generally four guttules, with a narrow sheath. *Sheath* drawn out 4–6 μm long at both ends, with a lateral pad, 1–1.5 μm wide at side. Asexual morph: undetermined.

Material examined: Thailand, Chiang Mai Province, on dead twigs attached to *Magnolia* sp. (*Magnoliaceae*), 13 September 2017, N.I. de Silva, NI189 (MFLU 18-1291, holotype).

GenBank numbers: LSU: ON870390; SSU: ON870915; ITS: ON878077.

Notes: According to the multi-gene phylogeny, *Leptoparies magnoliae* clustered with *L. palmarum* with 100% ML and 1.00 BYPP support (Figure 2). *Leptoparies magnoliae* can be distinguished from *L. palmarum* in having smaller asci and ascospores. *Leptoparies magnoliae* is characterized by the smaller asci (58 × 9 µm) and ascospores (16 × 5 µm), whereas *L. palmarum* is characterized by larger asci (93.9 × 11.9 μm) and ascospores (23.1 × 6.1 μm) [52]. Ascomata of *L. magnoliae* is smaller (240–290 µm high × 230–270 µm diameter) than *L. palmarum* (210–320 μm high and 490–650 μm diameter) [52]. Peridium of *L. magnoliae* is thinner 15–25 µm wide, comprising several layers of cells compared to *L. palmarum*, which is 25–32 μm wide, comprising 3–5 layers of cells [52]. A pairwise comparison of ITS sequence data between *L. magnoliae* and *L. palmarum* indicates 16 base pair (3.2%) differences across 500 nucleotides. It is interesting to note that *L. magnoliae* is the second species recorded for *Leptoparies*. The first species, *L. palmarum*, was identified by Hashimoto et al. [52] on petioles of *Trachycarpus fortunei* (*Arecaceae*) in Japan.

***Sulcatisporaceae*** Kaz. Tanaka and K. Hiray., Stud. Mycol. 82: 119 (2015)

Type: *Sulcatispora* Kaz. Tanaka and K. Hiray., Shirouzu and Hosoya, Stud. Mycol. 82: 120 (2015)

Notes: Tanaka et al. [53] established *Sulcatisporaceae* in *Pleosporales*. *Sulcatisporaceae* was typified by *Sulcatispora* Kaz. Tanaka and K. Hiray. and included two genera, *Magnicamarosporium* Kaz. Tanaka and K. Hiray. and *Neobambusicola* Crous and M.J. Wingf. [53]. The family is characterized by subglobose ascomata with a short ostiolar neck, trabeculate pseudoparaphyses, clavate asci, broadly fusiform ascospores, and ellipsoid to subglobose conidia with or without striate ornamentation [53]. Hongsanan et al. [18] accepted *Magnicamarosporium*, *Neobambusicola*, *Pseudobambusicola* Hern.-Restr. and Crous, and *Sulcatispora* in the family *Sulcatisporaceae*. Phukhamsakda et al. [36] added two more genera to the family viz. *Anthosulcatispora* Phukhams. and K.D. Hyde and *Parasulcatispora* Phukhams. and K.D. Hyde based on the combined dataset of LSU, ITS, SSU, and *tef1-α* sequence data and morphology. Recently, Wijayawardene et al. [54] added a genus, *Uniappendiculata*; thus, the number of genera in the family increased to seven. 

***Neobambusicola*** Crous and M.J. Wingf., Persoonia 33: 255 (2014)

Type: *Neobambusicola strelitziae* Crous and M.J. Wingf., Persoonia 33: 255 (2014)

Notes: *Neobambusicola* was introduced by Crous et al. [55] in *Bambusicolaceae*, *Pleosporales*. However, Tanaka et al. [53] transferred *Neobambusicola* to *Sulcatisporaceae* (*Pleosporales*) owing to the phylogenetic support with the two genera, *Sulcatispora* and *Magnicamarosporium*. Phookamsak et al. [47] introduced *N. brunnea* Chen and Norphanphoun based on phylogenetic analysis of a combined LSU and ITS dataset. However, *N. brunnea* was transferred to *Anthosulcatispora brunnea* (Chen and C. Norphanphoun) Phukhams. and K.D. Hyde by Phukhamsakda et al. [36]. The asexual morph of *Neobambusicola* is characterized by erumpent, globose conidiomata, subcylindrical to ampulliform, phialidic conidiogenous cells, hyaline, fusoid-ellipsoid, one-septate conidia, and microconidial state with doliiform to subcylindrical microconidiogenous cells and hyaline, subglobose to subcylindrical, aseptate microconidia [55]. Currently, the only known species of *Neobambusicola* is *N. strelitziae*, which was reported on the leaves of *Strelitzia nicolai* in South Africa [55]. We followed the latest treatment of Phukhamsakda et al. [36] for the current study.

***Neobambusicola magnoliae*** N.I. de Silva and S. Lumyong, sp. nov.

**Figure 8:**
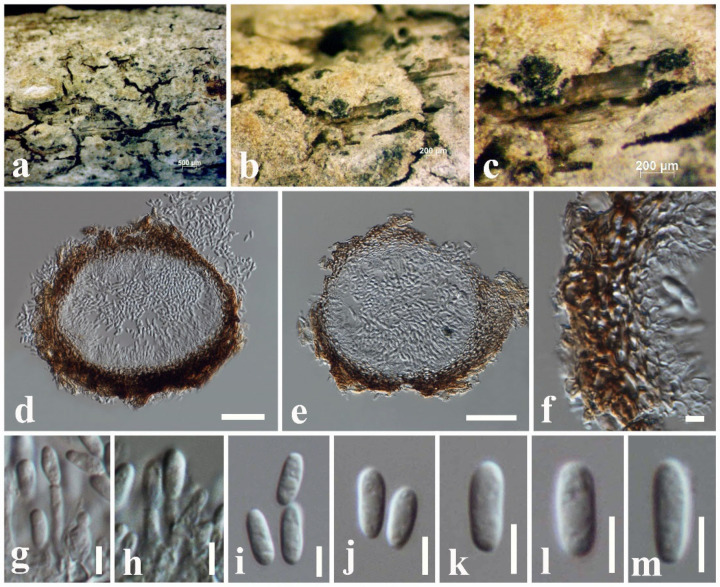
*Neobambusicola magnoliae* (HKAS 107122, holotype). (**a**–**c**) Appearance of conidiomata on substrate. (**d**,**e**) Vertical sections through conidiomata. (**f**) Conidiomatal wall. (**g**,**h**) Conidiogenous cells and developing conidia. (**i**–**m**) Conidia. Scale bars: (**c**) = 200 μm, (**d**,**e**) = 20 μm, (**f**–**m**) = 5 μm.

Index Fungorum number: IF559915, Faces of fungi number: FoF12710, Figure 8.

Etymology: Name reflects the host genus *Magnolia*, from which the new species was isolated.

Holotype: HKAS 107122

*Saprobic* on dead twigs attached to *Magnolia* sp. Sexual morph: undetermined. Asexual morph: Coelomycetous. *Conidiomata* 160–200 µm high × 180–230 µm diam., ($\bar{x}\;$= 175 × 190 µm, *n* = 10), dark brown to black, solitary or scattered, gregarious, unilocular, semi-immersed to immersed, globose to subglobose. *Conidiomatal wall* 20–25 μm wide, composed of several layers of small, flattened, thick-walled, brown to dark brown pseudoparenchymatous cells, arranged in a *textura angularis*, becoming thin-walled and lightly pigmented towards the inside. *Conidiophores* reduced to conidiogenous cells. *Conidiogenous cells* 7–12 × 1–3 µm ($\bar{x}$ = 9 × 2 μm, *n* = 10), hyaline, phialidic, cylindrical or ampulliform, integrated, hyaline, smooth-walled. *Conidia* 8–10 × 2–4 μm ($\bar{x}$ = 9 × 3 μm, *n* = 40), hyaline, oblong, subcylindrical, with granular content, both ends rounded, thin-walled.

Material examined: Thailand, Chiang Mai Province, dead twigs attached to *Magnolia* sp. (*Magnoliaceae*), 6 August 2019, N.I. de Silva, MGT53 (HKAS 107122, holotype).

GenBank numbers: LSU: ON870389; SSU: ON870914; ITS: ON878076; *tef1-α*: ON911576.

Notes: *Neobambusicola magnoliae* groups with the ex-type *N. strelitziae* (CBS 138869) with 95% ML, 1.00 BYPP statistical support (Figure 3). *Neobambusicola strelitziae* was introduced by Crous et al. [55] on leaves of *Strelitzia nicolai* (*Strelitziaceae*) from South Africa. They observed two types of conidia, hyaline, fusoid-ellipsoid, one-septate conidia and hyaline, subglobose to subcylindrical, aseptate microconidia [55]. However, *N. magnoliae* has one type of conidia. Conidia of *N. magnoliae* fits well with the microconidia of *N. strelitziae* in having hyaline, subglobose to subcylindrical, aseptate, granular, smooth and apex obtusely rounded [55]. However, the size of microconidia of *N. magnoliae* (8–10 × 2–4 μm) is larger than *N. strelitziae* (3–7 × 3–4 μm) [55]. A pairwise comparison of ITS sequence data between *N. magnoliae* and *N. strelitziae* indicates 60 base pair (12.7%) differences across 470 nucleotides. A pairwise comparison of *tef1-α* was not carried out, as *tef1-α* sequence data for *N. strelitziae* was not available in the GenBank. Due to these morphological and phylogenetic variations, we introduce *N. magnoliae* as novel species.

***Teichosporaceae*** M.E. Barr, Mycotaxon 82: 374 (2002)

Type: *Teichospora* Fuckel, Jb. nassau. Ver. Naturk. 23–24: 160(1870)

Notes: Barr [56] introduced *Teichosporaceae* to accommodate genera, namely *Bertiella* Sacc., *Byssothecium* Fuckel, *Chaetomastia* (Sacc.) Berl., *Immotthia* M.E. Barr, *Loculohypoxylon* M.E. Barr, *Moristroma* A.I. Romero and Samuels, *Sinodidymella* J.Z. Yue and O.E. Erikss., and *Teichospora* (type) based on morphological characteristics. A recent revision by Tennakoon et al. [37] accepted nine genera, namely *Asymmetrispora*, *Aurantiascoma* Thambug. and K.D. Hyde, *Floricola* Kohlm. and Volkm.-Kohlm., *Magnibotryascoma* Thambug. and K.D. Hyde, *Misturatosphaeria* Mugambi and Huhndorf, *Pseudoaurantiascoma* Thambug. and K.D. Hyde, *Pseudomisturatosphaeria* Thambug. abd K.D. Hyde, *Ramusculicola* Thambug. and K.D. Hyde, and *Teichospora* in *Teichosporaceae* with molecular data and three genera (*Chaetomastia* (Sacc.) Berl., *Loculohypoxylon* M.E. Barr and *Sinodidymella* J.Z. Yue and O.E. Erikss.) without molecular data in this family. Members of this family are saprobic on woody branches, herbaceous roots, rhizomes, bark or leaves [37,53]. The sexual morph is characterized by 4–8-spored, bitunicate, fissitunicate, cylindrical to oblong or subclavate asci and hyaline or brown, ellipsoid to oblong or fusiform, 1–3-septate or muriform ascospores with gelatinous sheath. The asexual morph is coelomycetous, characterized by hyaline, brown, cylindrical to elongate, aseptate or three-distoseptate conidia [18,37,51,57].

***Asymmetrispora*** Thambugala & K.D. Hyde

Type: *Asymmetrispora tennesseensis* (Mugambi, A.N. Mill. and Huhndorf) Thambug. and K.D. Hyde, Fungal Diversity: 50, (2015)

Notes: *Asymmetrispora* was introduced by Thambugala et al. [51] to accommodate two species, namely *A*. *tennesseensis* (type) and *A*. *mariae*, which were previously known as *Misturatosphaeria tennesseensis* and *M*. *mariae*, respectively. Subsequently, Jaklitsch et al. [57] synonymized this genus under *Teichospora* based on the broad genus concept. However, Tennakoon et al. [37] provided a comprehensive taxonomic revision for the family and re-established *Asymmetrispora* based on its distinct morphological variations and phylogenetic evidence. Currently, only two sexual morph species are accepted in this genus [48]. In this study, we introduce the first asexual morph species of the genus *Asymmetrispora*, namely *A. zingiberacearum* from dead stem of *H. coronarium* in the Taiwan Province of China.

***Asymmetrispora zingiberacearum*** Tennakoon and S. Lumyong, sp. nov.

**Figure 9:**
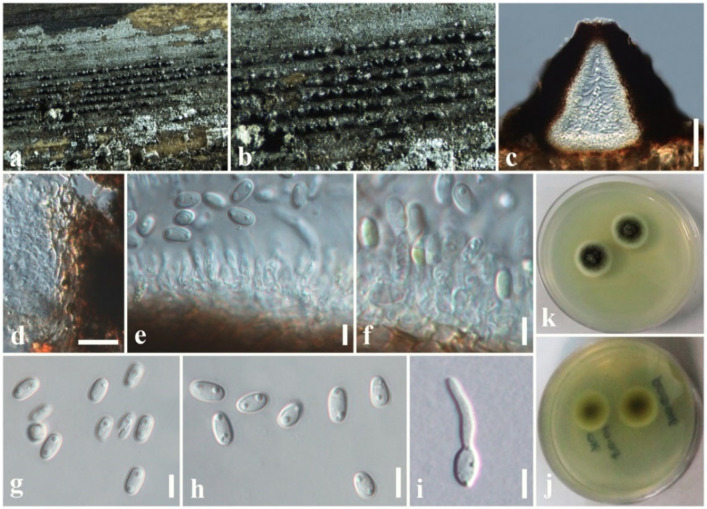
*Asymmetrispora zingiberacearum* (MFLU 19-2813, holotype). (**a**,**b**) Conidiomata on the host. (**c**) Section through conidioma. (**d**) Conidiomatal wall. (**e**,**f**) Conidiogenous cells and developing conidia. (**g**,**h**) Conidia. (**i**) A germinated conidium. (**j**) Colony from below (on PDA). (**k**) Colony from above (on PDA). Scale bars: (**c**) = 50 µm, (**d**) = 10 µm, (**e**–**i**) = 5 µm.

Index Fungorum Number: IF559916; Faces of fungi number: FoF12711, Figure 9.

Etymology: The species name reflects the host family *Zingiberaceae*, from which the holotype was collected.

Holotype: MFLU 19-2813

*Saprobic* on dead stem of *Hedychium coronarium*. Sexual morph: undetermined. Asexual morph: Coelomycetous. *Conidiomata* 100–170 µm high, 120–180 µm diam. ($\bar{x}\;$= 140 × 150 µm, *n* = 10), pycnidial, forming dark brown to black, linear, raised areas on the host surface, semi-immersed to erumpent, solitary or clustered, carbonaceous, globose to subglobose, ostiolate. *Conidiomatal wall* 10–20 μm wide, several layers of light brown, thick-walled of *textura angularis* cells, fusing at the outside indistinguishable from the host tissues. *Conidiophores* reduced to conidiogenous cells. *Conidiogenous cells* 5–10 × 3–5 μm ($\bar{x}$ = 8 × 4 µm, *n* = 20), hyaline, phialidic, cylindrical, slightly tapering towards apex and smooth-walled. *Conidia* 5–6 × 2–3 ($\bar{x}$ = 5.5 × 2.5 µm, *n* = 40) μm, aseptate, ellipsoid or obovoid obtuse ends, rarely cylindrical, initially hyaline, light brown at maturity, mostly two-guttulate, smooth-walled.

Culture characteristics: *Colonies* on PDA, 15–20 mm diam. after three weeks at 25 °C; colonies from above: circular, medium dense, slightly raised, surface smooth with entire edge, velvety appearance with smooth aspects, white to cream at the margin, grey in the center; reverse: yellowish brown at the margin, light brown in the center, mycelium white to whitish cream or grey.

Material examined: Taiwan Province of China, Chiayi, Ali Shan Mountain, Fanlu Township area, Dahu forest, on a dead stem of *Hedychium coronarium* (*Zingiberaceae*), 25 September 2018, D.S. Tennakoon, ROD016A (MFLU 19-2813, holotype); ex-type living culture, NCYUCC 19-0273. *ibid*. 28 September 2018, ROD016B (NCYU 19-0115, paratype); ex-paratype living culture, NCYUCC 19-0283.

GenBank numbers: (NCYUCC 19-0273) LSU: ON870391; ITS: ON878078; *tef1-α*: ON856444; (NCYUCC 19-0283) LSU: ON870392; ITS: ON878079; *tef1-α*: ON856445.

Notes: According to the multi-gene phylogenetic analyses (LSU, SSU, ITS, *tef1-α*, and *rpb2*), our strains (MFLU 19-2813 and NCYU 19-0115) grouped within *Asymmetrispora* isolates (*A*. *tennesseensis* and *A*. *mariae*) in a strongly supported clade (96% ML, 1.00 BYPP, Figure 4). Both *A. tennesseensis* and *A*. *mariae* species have been recorded as their sexual morphs. Thus, we could not compare the morphological differences between our new collection and *A. tennesseensis* and *A*. *mariae*. Therefore, we compared the ITS (+5.8 S) and *tef1-α* gene regions’ base pair differences. There are 10 base pair differences (1.96%) across 510 nucleotides across the ITS (+5.8 S) gene region and 26 base pair differences (3.59%) across 724 nucleotides across the *tef1-α* gene region between our collection (MFLU 19-2813) and *A. mariae* (CBS 140732). In addition, there are 29 base pair differences (3.86%) across 724 nucleotides across the *tef1-α* gene region between our collection (MFLU 19-2813) and *A. tennesseensis* (ANM 911). Therefore, we introduce our collection as a new species, *A. zingiberacearum*, from dead stems of *H. coronarium* (*Zingiberaceae*). It will be interesting to add fresh collections to expand this genus and to resolve the sexual and asexual connection of *Asymmetrispora* species.

***Ramusculicola*** Thambugala and K.D. Hyde, Fungal Divers 74: 249 (2015)

Type: *Ramusculicola thailandica* Thambug. and K.D. Hyde, Fungal Divers 74: 251 (2015)

Notes: Thambugala et al. [51] introduced *Ramusculicola* to accommodate the type *R. thailandica*. Species of *Ramusculicola* are saprobic on dead twigs [37]. The sexual morph is characterized by immersed ascomata, eight-spored, bitunicate, fissitunicate, cylindrical asci, hyaline, fusiform to cylindrical, usually 1–3-septate ascospores with thin mucilaginous sheath [51]. Two species were recorded for *Ramusculicola* in Index Fungorum [22].

***Ramusculicola thailandica*** Thambugala and K.D. Hyde, Fungal Divers 74: 251 (2015)

**Figure 10:**
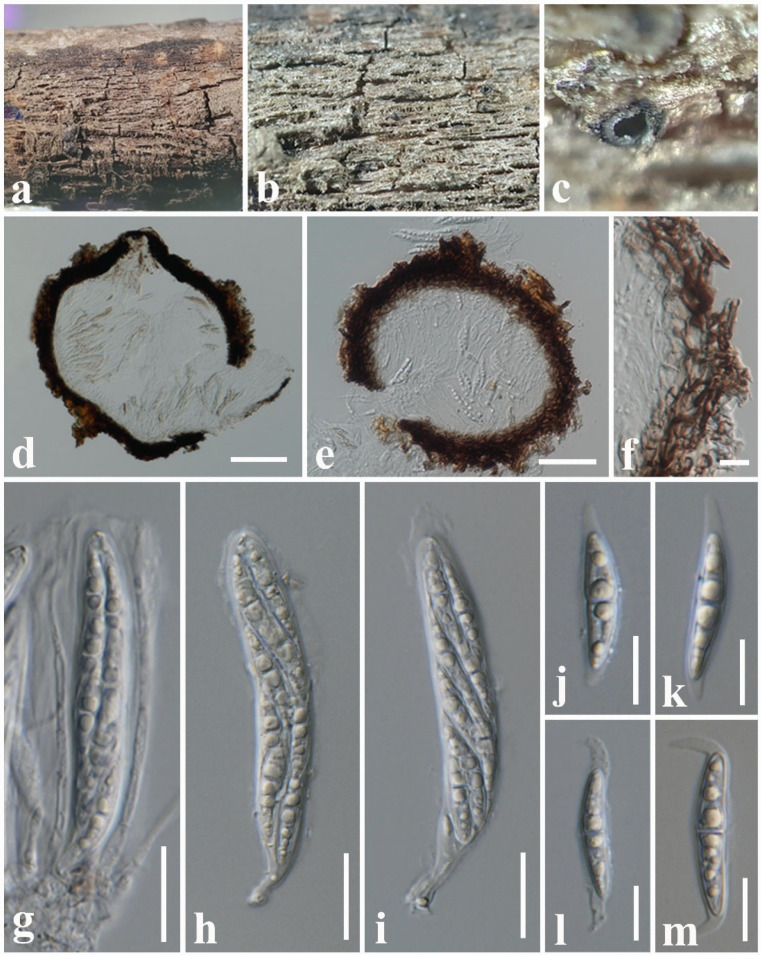
*Ramusculicola thailandica* (HKAS 107136, new host record). (**a**) The specimen. (**b**,**c**) Appearance of ascomata on substrate. (**d**,**e**) Vertical sections through ascoma. (**f**) Peridium. (**g**) Ascus and pseudoparaphyses. (**h**,**i**) Asci. (**j**–**m**) Ascospores. Scale bars: (**d**,**e**) = 50 μm, (**f**) = 10 μm, (**g**–**i**) = 20 μm, (**j**–**m**) = 10 μm.

Index Fungorum number: IF551265, Faces of fungi number: FoF 01092, Figure 10.

*Saprobic* on dead twigs attached to *Magnolia* sp. Sexual morph: *Ascomata* 180–200 µm high × 190–220 µm diam., ($\bar{x}$ = 190 × 200 µm, *n* = 10), dark brown to black, solitary or scattered, gregarious, unilocular, immersed, subglobose to globose, ostiolate. *Ostiole* central, rounded, compressed, periphysate, with a pore-like opening. *Peridium* 20–30 µm ($\bar{x}$ = 25 µm, *n* = 10), wide, 3–5 layers, composed of small, dark brown to black cells of *textura angularis*, fusing at the outside with the host tissues. *Hamathecium* comprising 2–3 μm wide, numerous, filamentous, indistinct septate, cellular pseudoparaphyses, anastomosing at the apex, embedded in a gelatinous matrix. *Asci* 80–100 × 11–15 µm ($\bar{x}$ = 90 × 13 µm, *n* = 20), eight-spored, bitunicate, fissitunicate, cylindrical, short pedicellate, apically rounded with an ocular chamber. *Ascospores* 18–27 × 3–6 µm ($\bar{x}$ = 20 × 4 µm, *n* = 30), overlapping, uniseriate, hyaline, fusiform to cylindrical or fusiform, usually one-septate, constricted at the septa, with small guttules, surrounded by a mucilaginous sheath. Asexual morph: undetermined.

Known hosts and distribution: Recorded from different host species in Thailand, including *Clematis sikkimensis* [51], *Ficus septica* [37], *Leucaena* sp. [58], and *Magnolia* sp. [this study].

Material examined: Thailand, Chiang Mai Province, dead twigs attached to *Magnolia* sp. (*Magnoliaceae*), 8 March 2019, N.I. de Silva, MGG4 (HKAS 107136).

GenBank numbers: LSU: ON870388; SSU: ON870913; ITS: ON878075; *tef1-α*: ON911577.

Notes: Our collection of HKAS 107136 clustered together with the ex-type of *Ramusculicola thailandica* (MFLUCC 13-0284) in the phylogeny of combined LSU, SSU, ITS, *tef1-α*, and *rpb2* sequence data (Figure 4). The morphological characteristics of the new collection (HKAS 107136) fit well with the type *R. thailandica* (MFLU 14-0587) in having immersed to erumpent, black, globose to subglobose, uni-loculate ascomata, cylindrical, short pedicellate asci and hyaline, fusiform to cylindrical, usually one-septate, guttulate ascospores surrounded by a mucilaginous sheath [51]. Further, the new collection also has a similar size range of asci and ascospores with the type *R. thailandica* (MFLU 14-0587). Therefore, we identified the new collection (HKAS 107136) as a new host record of *R. thailandica* from *Magnolia* species in Thailand.

***Dothideomycetes*** orders incertae sedis

***Kirschsteiniotheliales*** Hern.Restr. et al.

***Kirschsteiniotheliaceae*** Boonmee and K.D. Hyde, Mycologia 104(3): 705 (2012)

Type: *Kirschsteiniothelia* D. Hawksw., J. Linn. Soc., Bot. 91: 182 (1985)

Notes: *Kirschsteiniotheliaceae* was introduced by Boonmee et al. [59] to accommodate the genera *Kirschsteiniothelia* and *Dendryphiopsis* S. Hughes. *Dendryphiopsis* was considered the asexual morph of *Kirschsteiniothelia*. However, *Dendryphiopsis* was subsequently synonymized by *Kirschsteiniothelia*. Members of this family are widespread in the tropical regions and occur as saprobes on dead wood [42,59]. Sexual morph is characterized by superficial, subglobose to globose ascomata, bitunicate, fissitunicate asci, ellipsoidal, dull green, olive brown to dark brown, 1–2-septate, ascospores [59]. Asexual morph is characterized by straight to slightly curved, darkened conidiophores and broadly obovoid, fusiform to obclavate, reddish brown to dark brown, grayish brown, septate, conidia [59].

***Kirschsteiniothelia*** D. Hawksw., J. Linn. Soc., Bot. 91: 182 (1985)

Type: *Kirschsteiniothelia aethiops* (Sacc.) D. Hawksw., Bot. J. Linn. Soc. 91(1–2): 185 (1985)

Notes: *Kirschsteiniothelia* was introduced by Hawksworth [60] with the type species *K. aethiops*. Species of this genus are mainly saprobes on dead wood from terrestrial and aquatic habitats [18]. The sexual morph is superficial to semi-immersed, hemispherical or subglobose ascomata, bitunicate, eight-spored asci and brown to dark brown, ellipsoidal, smooth-walled, 1–2-septate ascospores [38,59]. The asexual morph is hyphomycetous with macronematous, mononematous, conidiophores, broadly ellipsoid-obovoid, brown to dark brown septate conidia [18]. Currently, 30 *Kirschsteiniothelia* species are listed in Species Fungorum [48].

***Kirschsteiniothelia thailandica*** Y.R. Sun, Yong Wang bis and K.D. Hyde, Phytotaxa 490(2): 175 (2021)

**Figure 11:**
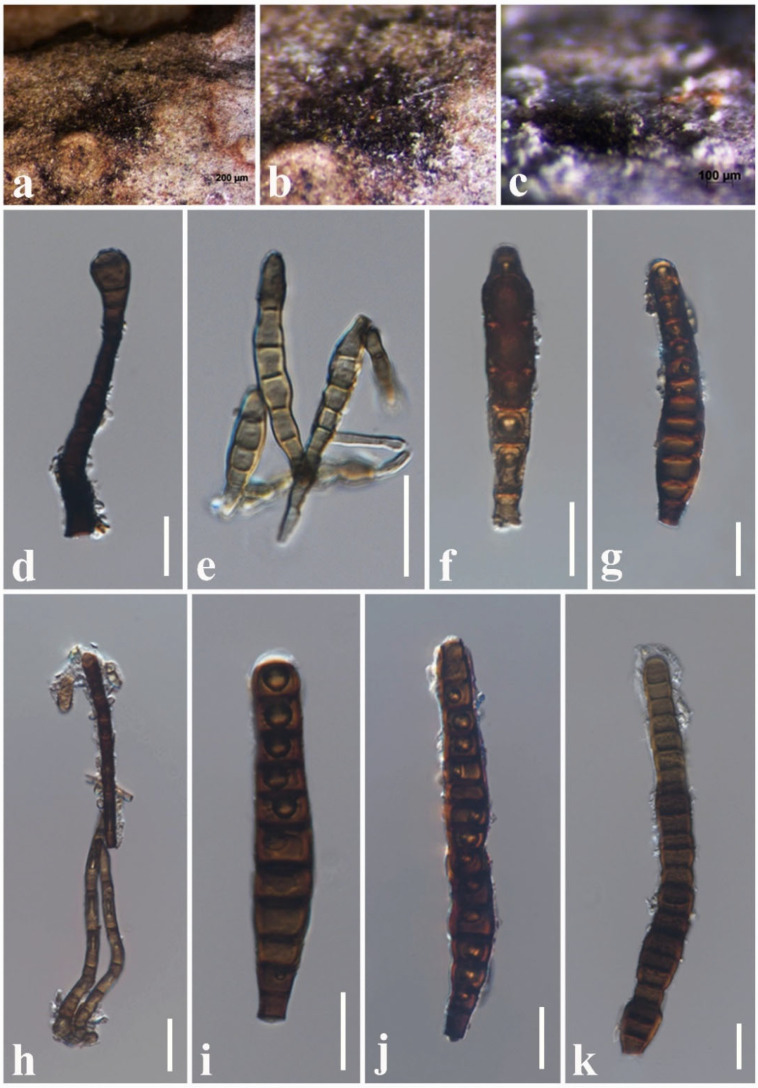
*Kirschsteiniothelia thailandica* (HKAS 107110, new host record). (**a**–**c**) Colonies on natural substrate. (**d**) Conidiophore and Conidiogenous cell. (**e**–**g**) Conidia. (**h**) Conidiophores. (**i**–**k**) Conidia. Scale bars: (**c**) = 100 μm, (**d**–**k**) = 20 μm.

Index Fungorum number: IF557949; Faces of fungi number: FoF 09289, Figure 11.

*Saprobic* on dead twigs attached to *Magnolia* sp. Sexual morph: undetermined. Asexual morph: Hyphomycetous. *Colonies* scattered, effuse, brown to dark brown, hairy on natural substrate. *Conidiophores* 60–95 × 8–12 μm ($\bar{x}$ = 76 × 10 µm, *n* = 10), macronematous, mononematous, brown to dark brown, solitary, erect, cylindrical, straight or slightly flexuous, unbranched, septate. *Conidiogenous cells* 10–24 × 8–10 μm ($\bar{x}$ = 17 × 9 µm, *n* = 10), brown to dark brown, cylindrical, monoblastic, integrated, terminal. *Conidia* 80–120 × 11–19 μm ($\bar{x}$ = 95 × 15 µm, *n* = 30), light brown to dark brown, obclavate, solitary, straight, acrogenous, 6–15-distoseptate, truncate at base, tapering towards apex, smooth-walled.

Culture characteristics: *Colonies* on PDA reaching 35 mm diam. after 1 week at 25 °C; colonies from above: circular, margin undulate, dense, slightly raised, cottony appearance, black at the margin, dark grey in the center; reverse: black.

Known hosts and distribution: from *Ficus macrocarpa* in Thailand [38] and *Magnolia* sp. in Thailand (this study).

Material examined: Thailand, Chiang Mai Province, dead twigs attached to *Magnolia* sp. (*Magnoliaceae*), 8 March 2019, N.I. de Silva, MGT15 (HKAS 107122), living culture, MFLUCC 22-0020.

GenBank numbers: LSU: ON870387; SSU: ON870912; ITS: ON878074.

Notes: *Kirschsteiniothelia thailandica* was introduced by Sun et al. [38] from dead twigs of *Ficus microcarpa* in Thailand. The morphological characteristics of our collection (HKAS 107122) resemble *K. thailandica* (MFLU 20–0263) in having monoblastic, integrated, terminal conidiogenous cells and brown, obclavate, straight, solitary, truncate at base, tapering towards apex, distoseptate conidia [38]. The type *K. thailandica* MFLU 20–0263 (74–110 × 13–20 μm) [38] and HKAS 107122 (80–120 × 11–19 μm) have an overlapping size range of conidia. According to the phylogeny, our strain MFLUCC 22-0020 nested with the ex-type *K. thailandica* (MFLUCC 20–0116) with 100% ML and 1.00 BYPP supports (Figure 5). Therefore, we report our collection as a new host record of *K. thailandica* from *Magnolia* species in Thailand.

## 4. Discussion

Studying the fungi in the tropics, particularly in Thailand and Taiwan Province of China, will provide important information towards establishing the numbers of fungi. The current study reveals three new species viz. *Asymmetrispora zingiberacearum* in Taiwan Province of China, *Leptoparies magnoliae* and *Neobambusicola magnoliae* in Thailand, and three new host records viz *Kirschsteiniothelia thailandica* and *Ramusculicola thailandica* in Thailand and *Sphaerellopsis paraphysata* in Taiwan Province of China. Considering the fact that tropical fungi are poorly documented and only a small percentage of global fungi have been discovered, which is between 2.6 and 4.5% of the 2.2–3.8 million estimated species, it is expected to reveal a large number of undiscovered taxa in the tropics. [7]. Thailand is geographically located in the core of the Greater Mekong Subregion with tropical seasonal forests and various types of floristic compositions [61,62]. Similarly, Taiwan Province of China has tropical to subtropical climatic zones that are attributed to the luxuriant vegetation, resulting in tremendous biodiversity [37]. These tropical regions have terrestrial ecosystems that have a great influence on regional and global energy and water cycling because these are located in regions of high solar radiation and evaporation [61]. Previous investigations have identified several novel fungal species and reported new host and geographical records in Thailand. For example, Doilom et al. [63] studied both asymptomatic stems and dead wood and symptomatic branches, stems, and leaves of *Tectona grandis* (*Lamiaceae*) in Thailand. Their investigation revealed 14 species and 14 new host and geographical records. In another study, Tibpromma et al. [64] identified seven new species and nine known species of endophytes associated with leaves of *Pandanaceae* collected from southern Thailand. Mapook et al. [65] introduced 12 new genera, 47 new species, and 12 new host records of microfungi associated with the invasive weed *Chromolaena odorata* collected in northern Thailand. In addition to these studies, Tennakoon et al. [66] explored microfungi associated with the leaf litter of different plants in Taiwan Province of China and revealed two new families, three new genera, 41 new species, and 54 new host records. These intensive studies generated important findings to support the fact that the tropics harbor enormous diversity of plant-associated microfungi and also provide a host-fungus database for future studies and increase knowledge of fungal diversity, as well as new fungal discovery.

*Dothideomycetes* are the largest and most ecologically diverse class of *Ascomycota*. Dothideomyceteous members exhibit as endophytes, pathogens, saprobes, or epiphytes on various hosts, in terrestrial as well as aquatic habitats [18,65]. We investigated saprobic *Dothideomycetes* on dead twigs and leaves from three different plant species, namely *Hedychium coronarium* and *Lilium longiflorum* and *Magnolia* species. In this study, we introduced *A. zingiberacearum* as new species on dead stems of *H. coronarium* (*Zingiberaceae*). Thambugala et al. [51] introduced the genus *Asymmetrispora* and incorporated two species, *A. tennesseensis* and *A. mariae*, that were previously known as *Misturatosphaeria tennesseensis* and *M. mariae*, respectively. Two species were accepted in *Asymmetrispora* in Species Fungorum [48]. *Asymmetrispora tennesseensis* was isolated from woody branches in the USA [67], while *A*. *mariae* was isolated from the bark and wood of *Robinia pseudoacacia* in Europe (Austria, France, and Germany) [57]. The novel species of *Asymmetrispora* introduced in this study, *A. zingiberacearum*, is also a saprobic species that was isolated from dead stems of *H. coronarium* in Taiwan Province of China. This indicates that *Asymmetrispora* are found in different geographical locations such as the USA, Europe (Austria, France, and Germany), and Taiwan Province of China. Another interesting fact is that both *A. tennesseensis* and *A*. *mariae* were described using their sexual morph characteristics. *Asymmetrispora zingiberacearum* is the first asexual morph (coelomycetous) that is recorded for this genus. The second host plant species investigated in the current study is *L. longiflorum*, a bulbous plant species of the Liliaceae, endemic to the Ryukyu archipelago and Taiwan [68]. *Lilium longiflorum* is regarded as an important species in world horticulture [68]. The current study reports *Sphaerellopsis paraphysata* from dead leaves of *L. longiflorum* (Liliaceae) in Taiwan Province of China for the first time. One of the findings here is that our collection slightly differs from the type of *S. paraphysata* in having smaller conidiomata and lacking paraphyses (Figure 6). This new information can be used to amend the morphology of *S. paraphysata*, which is useful for fungal identification.

*Magnolia* species are widely distributed in temperate and tropical Southeast and East Asia [69]. These plant species are important as ornamental plants due to their attractive flowers and foliage and are used as timber and medicine by local communities [69,70]. However, *Magnolia* species face habitat destruction, and 48% of all *Magnolia* species are considered endangered [71]. Hence, it is important to develop ex situ conservation for these endangered, endemic, and economically valuable plant species [71]. Microfungi on *Magnolia* species have been studied by various scientists around the world. According to Farr and Rossman [72], 1100 fungal taxa have been recorded from *Magnolia* species worldwide; however, 52 taxa have been reported in Thailand. In this study, we introduced two novel species, *Leptoparies magnoliae* (*Lophiostomataceae*) and *Neobambusicola magnoliae* (*Sulcatisporaceae*) from *Magnolia* species in Thailand. Both *Leptoparies* and *Neobambusicola* genera consist of only one known species to date [22]. *Leptoparies* was introduced by Hashimoto et al. [52] with the type species *Leptoparies palmarum*. *Neobambusicola* was introduced by Crous et al. [55] with the type species *N. strelitziae*. Further, *Kirschsteiniothelia thailandica* and *Ramusculicola thailandica* were reported herein as new host records from *Magnolia* species in Thailand. These novel findings demonstrate that *Magnolia* species are ideal candidates for studying microfungi, as they provide a suitable host environment for diverse microfungal occurrences. In addition, the current study added three new saprobic species as *A. zingiberacearum*, *L. magnoliae,* and *N. magnoliae* to the genera, *Asymmetrispora*, *Leptoparies* and *Neobambusicola*, respectively. Therefore, it will be interesting to explore and study fresh collections to uncover the hidden taxonomic diversity from the species-poor genera such as *Asymmetrispora*, *Leptoparies*, and *Neobambusicola*. Thus, these additional representative species will help to populate and better understanding of the genus.

Modern molecular techniques have exploded the ability to recognize fungal diversity and understand diverse fungal communities. In this study, the phylogeny of *Kirschsteiniothelia* was constructed using combined LSU, SSU, and ITS sequence data as provided in Boonmee et al. [59], Bao et al. [73], and Sun et al. [38]. Molecular phylogenetic studies were carried out mostly including LSU, SSU, mtSSU, and ITS, as well as the protein genes, such as *rpb1*, *rpb2*, *tef1-α*, *β-tubulin*, and ACT for *Pleosporales* taxa [15]. The use of a single molecular marker has not been successful in resolving numerous relationships. However, concatenated genes with additional protein-coding markers such as *tef1-α* and *rpb2* provide more precise phylogenetic affiliations of the members in *Dothideomycetes* [53,74]. Phylogenetic analyses of *Leptosphaeriaceae* were constructed using combined LSU, SSU, and ITS sequence data following the studies of Doilom et al. [33], Tennakoon et al. [75], and Wanasinghe et al. [76]. Since many strains of *Leptosphaeriaceae* lack *tef1-α* sequence data and other protein-coding markers, the phylogenetic studies were restricted to combined LSU, SSU, and ITS sequence data in accordance with Doilom et al. [33]. Generic delimitation in *Teichosporaceae* has been debatable in previous studies during the last decade because of insufficient taxon sampling and genetic marker coverage [37]. This species-rich family was erected by Barr [56] with eight genera, viz. *Bertiella*, *Byssothecium*, *Chaetomastia*, *Immotthia*, *Loculohypoxylon*, *Moristroma*, *Sinodidymella* and the genus type *Teichospora* based on morphological characteristics. The latest treatment of Tennakoon et al. [37] took advantage of both molecular and morphological approaches to delimitate species in *Teichosporaceae*. Their phylogenetic analyses based on a multigene-matrix of five genetic markers (ITS, LSU, SSU, *tef1-α*, and *rpb2*) and increased taxon sampling shed new light on the relationships of different genera within *Teichosporaceae*. However, the majority of strains lacked *rpb2* sequence data. It is, therefore, necessary to collect fresh samples and obtain molecular data including protein-coding markers such as *tef1-α* and *rpb2* for existing and novel species to investigate their phylogenetic relationships in order to achieve better identification and classification. Some of the more advanced molecular studies have examined a great amount of molecular data and identified fungal diversity at the generic level. Nevertheless, this results in the majority of uninformative data. Therefore, further studies should incorporate not only phylogenetic studies and morphological comparisons but also host associations and geographical information of microfungi.

## Data Availability

All sequences generated in this study were submitted to GenBank (https://www.ncbi.nlm.nih.gov, accessed on 1 October 2022).

## Supplementary Materials

The following supporting information can be downloaded at: https://www.mdpi.com/article/10.3390/jof8101094/s1, Table S1: GenBank and culture collection accession numbers of species included in this phylogenetic study (*Sphaerellopsis* tree). The newly generated sequences are shown in red.; Table S2: GenBank and culture collection accession numbers of species included in this phylogenetic study (*Leptoparies* tree). The newly generated sequences are shown in red.; Table S3: GenBank and culture collection accession numbers of species included in this phylogenetic study (*Neobambusicola* tree). The newly generated sequences are shown in red.; Table S4: GenBank and culture collection accession numbers of species included in this phylogenetic study (*Teichosporaceae* tree). The newly generated sequences are shown in red.; Table S5: GenBank and culture collection accession numbers of species included in this phylogenetic study (*Kirschsteiniothelia* tree). The newly generated sequences are shown in red.
