# Supplementary material for: Taxonomy and Phylogenetic Appraisal of Dothideomycetous Fungi Associated with Magnolia, Lilium longiflorum and Hedychium coronarium
Source: J Fungi (Basel). 2022 Oct 17;8(10):1094. doi: 10.3390/jof8101094 (PMC9604768; doi:10.3390/jof8101094)
Supplement: Supplementary file 1 [file jof-08-01094-s001.zip › jof-1895637-supplementary.pdf]

Supplementary Table S1. GenBank accession numbers and culture collection numbers of species of *Leptosphaeriaceae*. Ex-type strains are in bold. The newly generated sequence is indicated in red.

Species	Culture Collection / Voucher No.	GenBank Accession Numbers		
		LSU	SSU	ITS
Alloleptosphaeria italica	**MFLUCC 14-0934**	**KT454714**	**-**	**KT454722**
Alternariaster bidentis	**CBS 134021**	**KC609341**	**-**	**KC609333**
Alternariaster centaureae-diffusae	**MFLUCC 14-0992**	**KT454715**	**KT454730**	**KT454723**
Alternariaster helianthi	CBS 327.69	KC584369	KC584627	KC609335
Didymella exigua	**CBS 183.55**	**EU754155**	**EU754056**	**GU237794**
Heterosporicola chenopodii	CBS 115.96	EU754188	EU754089	JF740227
Heterosporicola dimorphospora	CBS 165.78	JF740281	JF740098	JF740204
Leptosphaeria cichorium	**MFLUCC 14-1063**	**KT454712**	**KT454728**	**KT454720**
Leptosphaeria conoidea	CBS 616.75	MH872726	JF740099	MH860957
Neoleptosphaeria rubefaciens	CBS 223.77	JF740312		JF740243
Ochraceocephala foeniculi	**CBS 145654**	**MN516774**	**MN516743**	**MN516753**
Paraleptosphaeria dryadis	CBS 643.86	GU301828	KC584632	JF740213
Paraleptosphaeria macrospora	CBS 114198	MH874520	-	MH862957
Paraphoma radicina	**CBS 111.79**	**EU754191**	**EU754092**	**FJ427058**
Phaeosphaeria oryzae	**CBS 110110**	**GQ387591**	**GQ387530**	**KF251186**
Plenodomus agnitus	CBS 121.89	JF740271	-	JF740194
Praeclarispora artemisiae	**KUMCC 20-0201A**	**MT957053**	**MT957046**	**MT957060**
Pseudoleptosphaeria etheridgei	**CBS 125980**	**JF740291**	**-**	**JF740221**
Querciphoma carteri	CBS 101633	GQ387593	GQ387532	KF251210
Sclerenchymomyces clematidis	**MFLUCC 17–2180**	**MT214558**	**MT226675**	**MT310605**
Sphaerellopsis artemisiae	**KUMCC 20-0202A**	**MT957058**	**MT957051**	**MT957065**
Sphaerellopsis artemisiae	KUMCC 20-0202B	MT957059	MT957052	MT957066
Sphaerellopsis filum	CBS 234.51	KP170723	-	KP170655
Sphaerellopsis filum	CBS 235.51	KP170724	-	KP170656
Sphaerellopsis filum	CBS 317.68	KP170725	-	KP170657
Sphaerellopsis hakeae	**CPC 29566T**	**KY173555**	**-**	**KY173466**
Sphaerellopsis isthmospora	**HKAS 102225A**	**MK387963**	**MK387933**	**MK387925**
Sphaerellopsis isthmospora	HKAS 102225B	MK387964	MK387934	MK387926
Sphaerellopsis macroconidialis	CBS 233.51	KP170726	-	KP170658
Sphaerellopsis macroconidialis	CBS 658.78	KP170727	-	KP170659
Sphaerellopsis macroconidialis	CPC 21113	KP170728	-	KP170660
Sphaerellopsis paraphysata	**CPC 21841**	**KP170729**	**-**	**KP170662**
Sphaerellopsis paraphysata	KUMCC 18-0195	MK387965	MK387935	MK387927
Sphaerellopsis paraphysata	**MFLU 19-2774**	**ON870393**	**ON870916**	**ON878080**
Subplenodomus apiicola	CBS 285.72	GU238040	GU238211	JF740196
Subplenodomus drobnjacensis	CBS 270.92	JF740286	-	JF740212

Supplementary Table S2. GenBank accession numbers and culture collection numbers of species of *Lophiostomataceae*. Ex-type strains are in bold. The newly generated sequence is indicated in red.

Species	Culture Collection/ Voucher No.	GenBank Accession Numbers				
		SSU	ITS	LSU	*tef1*	*rpb2*
Crassiclypeus aquaticus	KH 104	LC312470	LC312499	LC312528	LC312557	LC312586
Crassiclypeus aquaticus	**KT 970**	**LC312472**	**LC312501**	**LC312530**	**LC312559**	**LC312588**
Dimorphiopsis brachystegiae	**CPC 22679**	**-**	**KF777160**	**KF777213**	**-**	**-**
Flabellascoma aquaticum	**KUMCC 15-0258**	**-**	**MN304827**	**MN274564**	**MN328898**	**MN328895**
Flabellascoma cycadicola	**KT 2034**	**LC312473**	**LC312502**	**LC312531**	**LC312560**	**LC312589**
Flabellascoma fusiforme	**MFLUCC 18-1584**	**-**	**MN304830**	**MN274567**	**MN328902**	**-**
Flabellascoma minimum	**KT 2040**	**LC312475**	**LC312504**	**LC312533**	**LC312562**	**LC312591**
Flabellascoma minimum	KT 2013	LC312474	LC312503	LC312532	LC312561	LC312590
Lentistoma bipolare	KT 2415	LC312483	LC312513	LC312542	LC312571	LC312600
Lentistoma bipolare	**CBS 115375**	**LC312477**	**LC312506**	**LC312535**	**LC312564**	**LC312593**
Leptoparies palmarum	**KT 1653**	**LC312485**	**LC312514**	**LC312543**	**LC312572**	**LC312601**
Leptoparies magnoliae	**MFLU 18-1291**	**ON870915**	**ON878077**	**ON870390**	**-**	**-**
Lophiostoma clematidis	**MFLUCC 17-2081**	**MT226679**	**MN393004**	**MT214562**	**MT394741**	**MT394689**
Lophiostoma vitigenum	HH 26930	AB618697	LC001735	AB619015	LC001761	-
Lophiostoma vitigenum	HH 26931	AB618698	LC001736	AB619016	LC001762	-
Neovaginatispora clematidis	**MFLUCC 17-2156**	**MT226676**	**MT310606**	**MT214559**	**MT394738**	**-**
Neovaginatispora fuckelii	KT 634	AB618690	LC001732	AB619009	LC001750	-
Parapaucispora pseudoarmatispora	**KT 2237**	**LC100018**	**LC100021**	**LC100026**	**LC100030**	**-**
Paucispoar quadrispora	KH 448	LC001720	LC001733	LC001722	LC001754	-
Paucispoar quadrispora	**KT 843**	**AB618692**	**LC001734**	**AB619011**	**LC001755**	**-**
Paucispoar versicolor	**KH 110**	**LC001721**	**AB918731**	**AB918732**	**LC001760**	**-**
Pseudopaucispora brunneospora	**KH 227**	**LC312494**	**LC312523**	**LC312552**	**LC312581**	**LC312610**
Teichospora trabicola	C134	-	KU601591	KU601591	KU601601	KU601600
Vaginatispora amygdali	MFLUCC 18-1526	MK085057	MK085055	MK085059	MK087657	-
Vaginatispora amygdali	**KT 2248**	**LC312495**	**LC312524**	**LC312553**	**LC312582**	**LC312611**
Vaginatispora appendiculata	**MFLUCC 16-0314**	**KU743219**	**KU743217**	**KU743218**	**KU743220**	**-**
Vaginatispora aquatica	**MFLUCC 11-0083**	**KJ591575**	**KJ591577**	**KJ591576**	**-**	**-**
Vaginatispora armatispora	MFLUCC 18-0213	-	MN304826	MN274563	MN328897	MN328894
Vaginatispora armatispora	MFLUCC 18-0247	-	MK085056	MK085060	MK087658	MK087669
Vaginatispora microarmatispora	MTCC 12733	MF142594	MF142592	MF142593	MF142595	MF142596
Vaginatispora scabrispora	KT 2443	LC312496	LC312525	LC312554	LC312583	LC312612

Supplementary Table S3. GenBank accession numbers and culture collection numbers of species of *Sulcatisporaceae*. Ex-type strains are in bold. The newly generated sequence is indicated in red.

Species	Culture Collection / Voucher No.	GenBank Accession Numbers			
		LSU	SSU	ITS	TEF
Anthosulcatispora subglobosa	MFLUCC 17–2065	MT214592	MT226705	MT310636	MT394649
Bambusicola bambusae	MFLUCC 11-0614	JX442035	JX442039	NR_121546	-
Bambusicola massarinia	**MFLUCC 11-0389**	**JX442037**	**JX442041**	**NR_121548**	**-**
Bambusicola pustulata	MFLUCC 15-0190	KU863107	KU872112	KU940118	KU940190
Bambusicola splendida	MFLUCC 11-0439	JX442038	JX442042	NR_121549	-
Bambusicola triseptatispora	MFLUCC 11-0166	KU863109	-	KU940120	-
Didymosphaeria rubi-ulmifolii	MFLUCC 14-0024	KJ436585	KJ436587	-	-
Latorua caligans	**CBS 576.65**	**KR873266**	**-**	**NR_132923**	**-**
Latorua grootfonteinensis	CBS 369.72	KR873267	-	-	-
Magnicamarosporium diospyricola	**MFLUCC 16-0419**	KY554212	KY554211	KY554210	KY554209
Magnicamarosporium iriomotense	**KT 2822**	**AB807509**	**AB797219**	**AB809640**	**AB808485**
Neobambusicola brunnea	**MFLU 18-1393**	**MH644791**	**-**	**MH644792**	**-**
Neobambusicola strelitziae	**CBS 138869**	**KP004495**	**-**	**KP004467**	**-**
Neobambusicola magnoliae	**HKAS 107122**	**ON870389**	**ON870914**	**ON878076**	**ON911576**
Parasulcatispora clematidis	MFLUCC 17–2082	MT214593	-	MT310637	MT394650
Pseudobambusicola thailandica	BCC 79462	MG926560	MG926559	-	MG926562
Sulcatispora acerina	KT 2982T	LC014610	LC014605	LC014597	LC014615
Sulcatispora berchemiae	KT 1607	AB807534	AB797244	AB809635	AB808509

Supplementary Table S4. GenBank accession numbers and culture collection numbers of species of *Teichosporaceae*. Ex-type strains are in bold. The newly generated sequence is indicated in red.

Species	Culture Collection/ Voucher No.	GenBank Accession Numbers			
		LSU	ITS	SSU	*tef1*
Angustimassarina acerina	**MFLUCC 14-0505**	**KP888637**	**KP899132**	**KP899123**	**KR075168**
Angustimassarina populi	**MFLUCC 13-0034**	**KP888642**	**KP899137**	**KP899128**	**MG812528**
Angustimassarina quercicola	**MFLUCC 14-0506**	**KP888638**	**KP899133**	**KP899124**	**KR075169**
Asymmetrispora mariae	C136	-	KU601583	-	KU601613
Asymmetrispora mariae	**CBS 140732**	**KU601580**	**KU601580**	**-**	**KU601614**
Asymmetrispora mariae	C 139	KU601582	KU601582	-	KU601615
Asymmetrispora mariae	C 136	KU601581	KU601581	-	KU601611
Asymmetrispora tennesseensis	**ANM 911**	**GU385207**	**-**	**-**	**GU327769**
Asymmetrispora zingiberacearum	**NCYUCC 19-0273**	**ON870391**	**ON878078**	**-**	**ON856444**
Asymmetrispora zingiberacearum	NCYUCC 19-0283	ON870392	ON878079	-	ON856445
Aurantiascoma parva	ANM 60	GU385182	-	-	-
Aurantiascoma parva	**GKM 169N**	**GU385165**	**-**	**-**	**GU327768**
Aurantiascoma quercus	**CBS 143396**	**MH107966**	**MH107920**	**-**	**MH108030**
Decaisnella formosa	BCC 25617	GQ925847	-	GQ925834	GU479850

Decaisnella formosa	BCC 25616	GQ925846	-	GQ925833	GU479851
Floricola clematidis	**MFLUCC 17–2182**	**MT214594**	**MT310638**	**MT226706**	**MT394651**
Floricola festucae	**MFLU 17-0773**	**MW587275**	**MW587280**	**MW587277**	**MW590256**
Floricola striata	**JK 5603K**	**GU479785**	**-**	**GU479751**	**-**
Floricola striata	JK 5678I	GU301813	-	GU296149	GU479852
Floricola viticola	**MFLUCC 15-0039**	**KT305993**	**KT305997**	**KT305995**	**-**
Guttulispora crataegi	MFLUCC 14-0993	KP888640	KP899135	KP899126	KR075162
Halotthia posidoniae	BBH 22481	GU479786	-	GU479752	-
Hermatomyces tectonae	**MFLUCC 14-1140**	**KU764695**	**KU144917**	**KU712465**	**-**
Hermatomyces thailandica	**MFLUCC 14-1143**	**KU764692**	**KU144920**	**KU712468**	**-**
Lophiostoma arundinis	JCM 13550	AB618998	JN942964	AB618679	LC001737
Lophiostoma arundinis	AFTOL-ID 1606	DQ782384	-	DQ782383	DQ782387
Lophiostoma crenatum	AFTOL-ID 1581	DQ678069		DQ678017	DQ677912
Magnibotryascoma acaciae	CPC 24801	KR611898	KR611877	-	-
Magnibotryascoma mali	MFLUCC 17-0933	MF173429	MF173433	MF173431	MF173435
Magnibotryascoma melanommoides	MP5	-	KU601585	-	KU601610
Mauritiana rhizophorae	BCC 28866	GU371824		GU371832	GU371817
Misturatosphaeria aurantiacinotata	GKM 1280	GU385174	-	-	GU327762
Misturatosphaeria aurantiacinotata	**GKM 1238**	**GU385173**	**-**	**-**	**GU327761**
Misturatosphaeria viridibrunnea	MFLUCC 10-0930	MH940252	MH940251	-	-
Paulkirkia arundinis	**MFLU 13-0315**	**-**	**-**	**-**	**-**
Pseudoaurantiascoma kenyensis	**GKM 1195**	**GU385194**	**-**	**-**	**GU327767**
Pseudoaurantiascoma kenyensis	GKM 234N	GU385188	-	-	GU327765
Pseudoaurantiascoma kenyensis	GKM L100Na	GU385189	-	-	GU327766
Pseudoaurantiascoma kenyensis	GKM 194N	-	-	-	GU327764
Pseudomisturatosphaeria cruciformis	**SMH 5151**	**GU385211**	**-**	**-**	**-**
Ramusculicola clematidis	**MFLUCC 17-2146**	**MT214596**	**MT310640**	**MT226707**	**MT394652**
Ramusculicola thailandica	MFLUCC 17-0884		MW587274	MW590255	MW587279
Ramusculicola thailandica	**MFLUCC 13-0284**	**KP888647**	**KP899141**	**KP899131**	**KR075167**
Ramusculicola thailandica	MFLUCC 10-0126	KP888644	KP899138	KP899130	KR075170
Ramusculicola thailandica	HKAS 107136	ON870388	ON878075	ON870913	ON911577
Sigarispora arundinis	KT651	-	-	-	LC001738
Sporormiella minima	S:Lundqvist 17212-a	GQ203744	GQ203786	-	-
Sulcosporium thailandica	MFLUCC 12-0004	-	MG520958	-	-
Teichospora austroafricana	**CBS 119330**	**EU552115**	**EU552115**	**-**	**-**
Teichospora austroafricana	CBS 122674	EU552116	EU552116	-	-
Teichospora trabicola	C 141	KU601592	KU601592	-	KU601603
Teichospora trabicola	C 157	KU601593	KU601593	-	KU601604
Teichospora trabicola	C 160	KU601594	KU601594	-	KU601602
Teichospora trabicola	**CBS 140730**	**KU601591**	**KU601591**	**-**	**KU601600**
Westerdykella cylindrica	AFTOL-ID 1037	-	DQ491519	-	DQ497610
Westerdykella ornata	CBS 379.55	GU301880	-	GU296208	GU349021

Supplementary Table S5. GenBank accession numbers and culture collection numbers of *Kirschsteiniothelia* species. Ex-type strains are in bold. The newly generated sequence is indicated in red.

Species	Culture Collection/ Voucher No.	GenBank Accession Numbers		
		ITS	LSU	SSU
Kirschsteiniothelia aethiops	CBS 109.53	-	AY016361	AY016344
Kirschsteiniothelia aethiops	MFLUCC 15–0424	KU500571	KU500578	KU500585
Kirschsteiniothelia aquatica	MFLUCC 17–1685	MH182587	MH182594	MH182618
Kirschsteiniothelia arasbaranica	IRAN 2509C	KX621986	KX621987	KX621988
Kirschsteiniothelia arasbaranica	IRAN 2508C	KX621983	KX621984	KX621985
Kirschsteiniothelia cangshanensis	MFLUCC 16–1350	MH182584	MH182592	-
Kirschsteiniothelia fluminicola	MFLUCC 16–1263	MH182582	MH182588	-
Kirschsteiniothelia lignicola	MFLUCC 10–0036	HQ441567	HQ441568	HQ441569
Kirschsteiniothelia phoenicis	MFLUCC 18–0216	MG859978	MG860484	MG859979
Kirschsteiniothelia rostrata	MFLUCC 15–0619	KY697280	KY697276	KY697278
Kirschsteiniothelia rostrata	MFLUCC 16–1124	-	MH182590	-
Kirschsteiniothelia submersa	MFLUCC 15–0427	KU500570	KU500577	KU500584
Kirschsteiniothelia submersa	S–481	-	MH182591	MH182616
Kirschsteiniothelia submersa	S–601	MH182585	MH182593	-
Kirschsteiniothelia tectonae	MFLUCC 12–0050	KU144916	KU764707	-
Kirschsteiniothelia thailandica	MFLUCC 20–0116	MT985633	MT984443	MT984280
Kirschsteiniothelia thailandica	MFLUCC 22-0020	ON878074	ON870387	ON870912
Kirschsteiniothelia thujina	JF 13210	KM982716	KM982718	KM982717
Phyllobathelium anomalum	MPN 242	-	GU327722	JN887386
Phyllobathelium firmum	ERP 3175	-	GU327723	-
Pseudorobillarda eucalypti	MFLUCC 12–0422	KF827451	KF827457	KF827463
Pseudorobillarda phragmitis	CBS 398.61	MH858101	EU754203	EU754104
Strigula guangxiensis	HMAS-L0138040	NR146255	MK206256	-
Strigula univelbiserialis	HMAS-L0137657	-	MK206243	MK206224
